# Niche differentiation of bacterial communities at a millimeter scale in Shark Bay microbial mats

**DOI:** 10.1038/srep15607

**Published:** 2015-10-26

**Authors:** Hon Lun Wong, Daniela-Lee Smith, Pieter T. Visscher, Brendan P. Burns

**Affiliations:** 1 School of Biotechnology and Biomolecular Sciences, The University of New South Wales, Sydney, Australia; 2 Department of Marine Sciences, University of Connecticut, USA; 3 Australian Centre for Astrobiology, University of New South Wales Sydney, Australia

## Abstract

Modern microbial mats can provide key insights into early Earth ecosystems, and Shark Bay, Australia, holds one of the best examples of these systems. Identifying the spatial distribution of microorganisms with mat depth facilitates a greater understanding of specific niches and potentially novel microbial interactions. High throughput sequencing coupled with elemental analyses and biogeochemical measurements of two distinct mat types (smooth and pustular) at a millimeter scale were undertaken in the present study. A total of 8,263,982 16S rRNA gene sequences were obtained, which were affiliated to 58 bacterial and candidate phyla. The surface of both mats were dominated by Cyanobacteria, accompanied with known or putative members of Alphaproteobacteria and Bacteroidetes. The deeper anoxic layers of smooth mats were dominated by Chloroflexi, while Alphaproteobacteria dominated the lower layers of pustular mats. *In situ* microelectrode measurements revealed smooth mats have a steeper profile of O_2_ and H_2_S concentrations, as well as higher oxygen production, consumption, and sulfate reduction rates. Specific elements (Mo, Mg, Mn, Fe, V, P) could be correlated with specific mat types and putative phylogenetic groups. Models are proposed for these systems suggesting putative surface anoxic niches, differential nitrogen fixing niches, and those coupled with methane metabolism.

Stromatolites are believed to represent one of the first microbial ecosystems, and may have had major impacts on past global biogeochemical cycles, particularly oxygen, nitrogen, hydrogen and sulfur^1^. Fossil evidence suggests they have been present on Earth, at varied levels of distribution, for more than 3 billion years^2^. Modern microbial mats are considered excellent analogues to these extinct geobiological formations^3^, and form in a range of environments, including the hypersaline settings of Guerrero Negro, Mexico^4^, and Hamelin Pool, Australia^5^,^6^. Hamelin Pool, in Shark Bay, harbors some of the most extensive marine microbialite systems, with ca. 300 km^2^ of both lithified and non-lithified formations^7^. The systems in Shark Bay are subjected to a range of stresses that likely shape the microbial communities, including high salinity, UV, and desiccation^7^,^8^.

The classical view of microbial mat systems is that they are visibly stratified miniature ‘living laboratories’, where a network of stratified, microbial metabolisms, interact to take advantage of abiotic and biotic factors^3^. The relationships between microbial metabolisms results in linked redox reactions, leading to the efficient cycling of nutrients throughout the mat^9^,^10^. The result is sharp gradients of different solute concentrations at different depths, creating microenvironments at a millimeter scale, which subsequently cause metabolic heterogeneity throughout the mat^11^,^12^,^13^. Consequently, a wide range of metabolic activities may impose steep chemical gradients and create niches with fine spatial resolution^10^,^14^. Microbial interactions, for instance, the exchange of certain nutrients, may determine the presence of certain niches within a mat. Recent studies in several microbial mats systems have examined solute distribution over a diel cycle^15^,^16^,^17^, with the finding of high rates of sulfate reduction occurring alongside oxygenic photosynthesis in Shark Bay^15^. These findings are part of a changing paradigm regarding sulfate reduction, in which evidence is suggesting what was thought to be an anaerobic process, actually occurs, and is tolerant of high oxygen conditions^18^,^19^,^20^. These findings raise open questions on the presence of specific niches in these systems (e.g. putative anaerobic niches in oxic layers) and illustrate that current knowledge regarding microbial community structure may still not be completely understood^21^,^22^.

A recent study on the Guerrero Negro mats has provided detailed information at the millimeter scale on microbial community composition determined by high throughput 16S rDNA sequencing^13^. Results showed that these hypersaline mats are phylogenetically stratified, with the most variation at the photic zone and oxic/anoxic transition interface. The Guerrero Negro mats were highly structured with depth^13^, which may be indicative of niche differentiation throughout different depths of the mat. However to date comparative data on microbial structure at such a scale is lacking in the extensive Shark Bay systems.

Previous published studies examining microbial diversity in Shark Bay microbial mats have focused primarily on the diversity present over a total mat depth of ca. 2 cm, with the communities dominated by Alphaproteobacteria and Bacteroidetes, and a lower than expected abundance of cyanobacteria^7^,^8^,^23^. These analyses may be however limited with respect to spatial distribution, and thus the aim of the present study was to undertake for the first time analyses of microbial community structure at a discrete millimeter scale in a lithifying (smooth) and non-lithifying (pustular) microbial mat in Shark Bay. High throughput sequencing of small subunit RNA (using a MiSeq Illumina platform), coupled with elemental analyses and biogeochemical measurements of the two distinct mat types were undertaken. The combination in this study of a high throughput sequencing approach with corresponding elemental profiles, has significantly enhanced our understanding of putative microbial niches and functional roles of different bacterial groups in these ecosystems.

## Results

### General statistics

**Figure 1:**
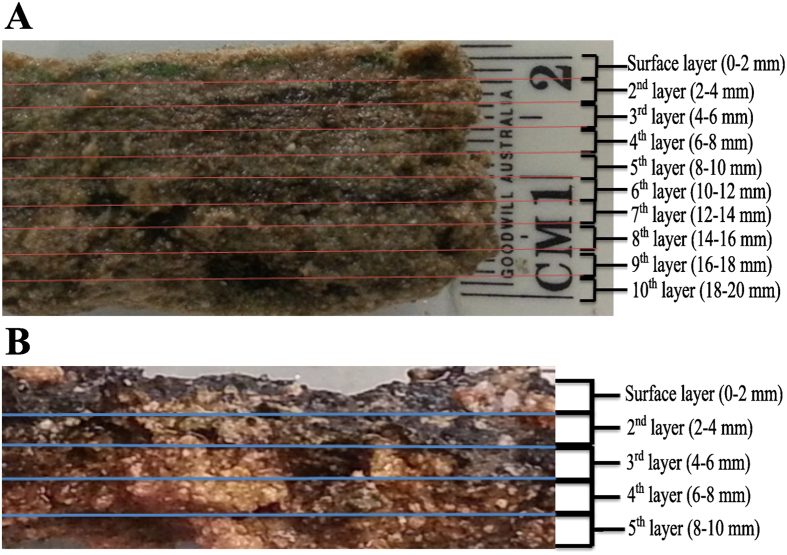
Vertical cross section of Shark Bay microbial mats indicating layers analysed. (**A**) Smooth mat sample of 2 cm vertical depth, dissected into ten 2 mm-layers. (**B**) Pustular mat sample of 1 cm vertical depth, dissected into five 2 mm-layers. Lines indicate the line of sectioning.

Microbial mats were analysed at discrete depths at two millimetre intervals (Fig. 1, details in Materials and Methods). To delineate for the first time the bacterial community composition among the different layers at depth in distinct Shark Bay microbial mats, high throughput Illumina MiSeq paired end sequencing was conducted. Using the bacterial V1–V3 primers, a total of 8,263,982 16S rRNA gene sequences sequences were obtained. Chimeric reads were removed, as such reads can be falsely interpreted as novel organisms, and thus can inflate and distort apparent microbial diversity. To account for sampling bias, the databases were subsequently subsampled so that each sample contained 50,000 sequences, with a total of 1,750,000 seqeuences in all the samples. The classified sequences were affiliated to 58 bacterial and candidate phyla, indicating a large diversity covering a wide range of bacterial phyla. Dominant bacterial groups that demonstrated more than 0.5% abundance in any layer were affiliated to 14 bacterial phyla and nine candidate divisions in smooth mats, while only 12 bacterial phyla and two candidate divisions were observed in pustular mats.

### Estimation of richness and diversity

In order to determine bacterial diversity and richness, rarefaction analysis and non-parametric estimators were performed. Alpha diversity analysis of all samples were performed at 3% genetic divergence level of OTUs. Rarefaction analysis indicated that curves start to level off but did not yet reach an asymptote (Supplementary Fig. S1), while non parametric estimators revealed that the observed number of OTUs range from 3671 (smooth mats layer 5) to 6646 (smooth mats layer 10). Smooth mats have an average of 4818 observed number of OTUs while pustular mats have 4636. In general, bacterial diversity increased with depth in smooth mats, except for layer 6 (depth 11–12 mm) where there was a sharp increase in Shannon diversity index (Supplementary Table 1). For pustular mats, bacterial diversity remained relatively the same throughout the depths. Shannon indices ranged from 5.47 to 6.77 in smooth mats whereas a narrower range of 6.06 to 6.29 was observed in pustular mats. In smooth mats, the surface layer has the lowest diversity, whilst at the bottom layer (18–20 mm) the largest bacterial diversity was observed. In pustular mats, the surface layer has the highest diversity while the subsequent layer (2–4 mm) was the least diverse.

### Phylogenetic composition and microelectrode measurements with depth

**Figure 2:**
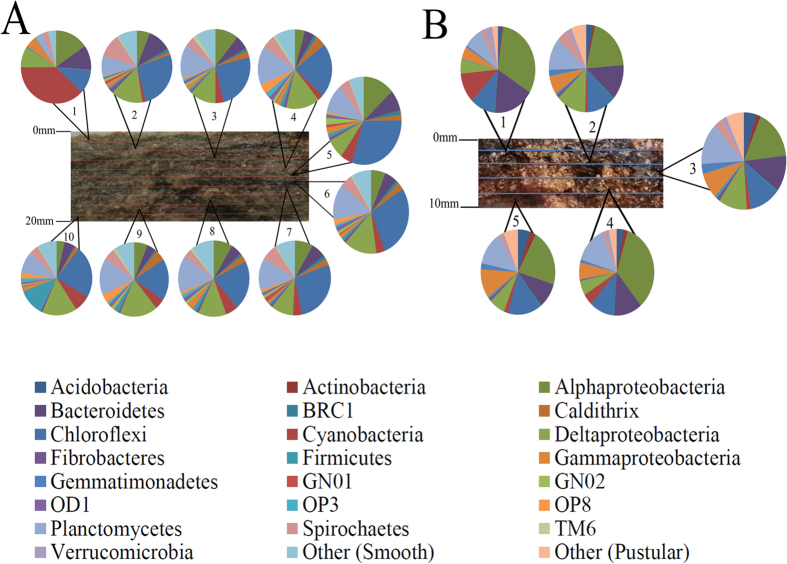
Bacterial phyla distribution in Shark Bay microbial mats at 2 mm depth intervals. (**A**) Bacterial phyla and proteobacterial classes found in different depths in smooth mats. Bacterial phyla with a relative abundance lower than 0.5% were classified into the group ‘Other’, that includes Acidobacteria, Actinobacteria, Gemmatimonadetes, Verrucomicrobia, Lentisphaerae, Chlorobi, SAR406, WS1, WS3, KSB3, Hyd24-12, OP9, Aquificae, LCP-89, FCPU426, Armatimonadetes, Tenericutes. (**B**) Bacterial phyla and proteobacterial classes found in different depths in pustular mats. Bacterial phyla with a relative abundance lower than 0.5% were classified into the group ‘Other’ that includes, Lentisphaerae, Chlorobi, SAR406, GN02, NKB19, Nitrospirae, PAUC34f, OD1, TM6, Dictyoglomi and SR1.

**Figure 3:**
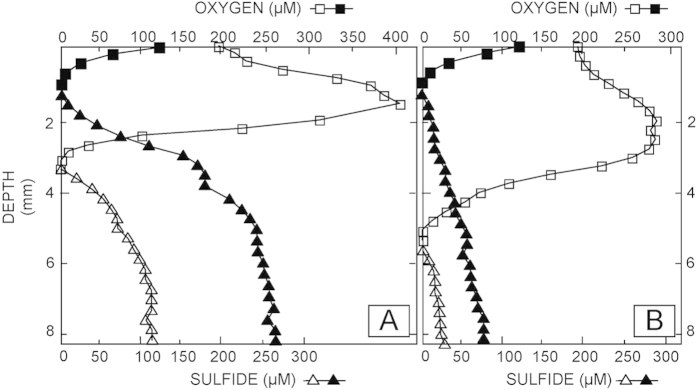
*In situ* depth profiles of oxygen and sulfide concentrations in Shark Bay microbial mats. Oxygen and sulfide concentrations were measured during peak photosynthesis between noon and 2:00pm (open symbols) and the end of the night, between 4:00 and 5:00am (closed/filled symbols). Oxygen and sulfide electrodes were deployed within 1.5 mm from each other. Multiple profiles (n = 3–7) were measured and representative profiles are shown. Squares represent oxygen, triangles sulfide concentrations. (**A**) Smooth mat. The oxygen concentration peaks at the subsurface layer of smooth mats during daytime (1–2 mm), but is zero below 4 mm. The sulfide concentration increases from 3 mm to 8 mm, with lower concentration during the day compared to night. (**B**) Pustular mat. The maximum oxygen concentration is found at the subsurface layer of pustular mats during daytime (1–2 mm). Permanent anoxic conditions prevail below 5 mm. The sulfide concentration is first observed at 5 mm, increases with increasing depth and has a lower concentration during the day compared to night.

Results of bacterial composition of both mats revealed phylogenetic stratification even at the phylum level (Fig. 2). Cyanobacteria, Proteobacteria and Bacteroidetes dominated the surface layer (0–2 mm) in both mat types. Phylum distribution became more uniform with depth, although subphylum variation was observed in successive layers. *In situ* microelectrode measurements of oxygen and sulfide with depth at the time of sampling suggest patterns of metabolic activity that can be linked to putative phylogenetic groups (Fig. 3). These depth profiles represent the combined effect of production and consumption processes (e.g., oxygenic photosynthesis as O_2_ sources vs. aerobic respiration, aerobic sulfide oxidation and chemical oxidation reactions as O_2_ sinks). To further explore the main metabolic activities, we performed light-dark shift experiments.

**Figure 4:**
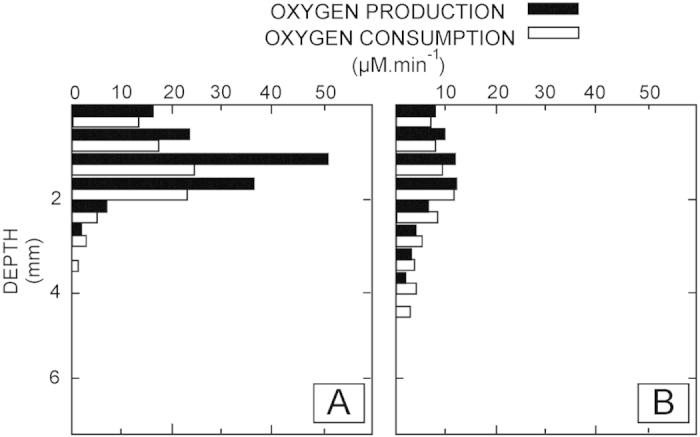
Depth profiles of oxygen production and consumption in the Shark Bay microbial mats. Oxygen production and consumption were measured *ex situ* using the light-dark shift technique. Increase in [O_2_] in the light is a function of production (i.e., photosynthesis) and consumption (i.e., predominantly aerobic respiration, chemolithotrophic sulfide oxidation and chemical reactions). (**A**) Smooth mat, which show high rates of production (closed bars) and consumption (open bars) in a shallow surface horizon. (**B**) Pustular mat, showing low rates of O_2_ production and consumption that prevail down to ca. 5.5 mm depth.

The photic zone was represented by the surface layer (0–2 mm in smooth mats, 0–3.5 mm in pustular mats), as oxygen concentrations and production rates reached a maximum during day time and the majority of cyanobacteria reside here. Oxygen concentrations and consumption rates diminished and were not detected below 3 and 5 mm during the day in smooth and pustular mats, respectively (Fig. 4). The steep O_2_ gradients and high production and consumption rates reflect very high metabolic rates of aerobes and thus intense cycling of carbon in the upper layers of smooth mats in particular. The steeper gradients and higher rates observed in smooth mats (Fig. 3a) when compared to pustular mats suggest a higher cell density (or conversely, a smaller amount of exopolymeric subtances) and a higher potential of carbonate precipitation^16^,^24^,^25^. Indeed, the light-dark shift experiments revealed a high rate of O_2_ production (i.e., oxygenic photosynthesis) and slightly lower rate of O_2_ consumption (i.e., predominantly aerobic respiration) (Fig. 4). Combining the balance of O_2_ production and consumption with the stiochiometric coupling of O and C production (i.e., CO_2_ + H_2_O → [CH_2_O] + O_2_)^12^; reveals a surplus of C available in the surface of the mat. This is corroborated with the O_2_ profiles, which indicate a flux of oxygen out of the mat during the daytime. This surplus provides a substrate for anaerobic metabolisms, such as fermentation, sulfate reduction, and methanogenesis^21^ which appear to prevail in the oxic zone of microbial mats. Conversely at night, oxygen was rapidly depleted in the first 1 mm of the mats (Fig. 3).

**Figure 5:**
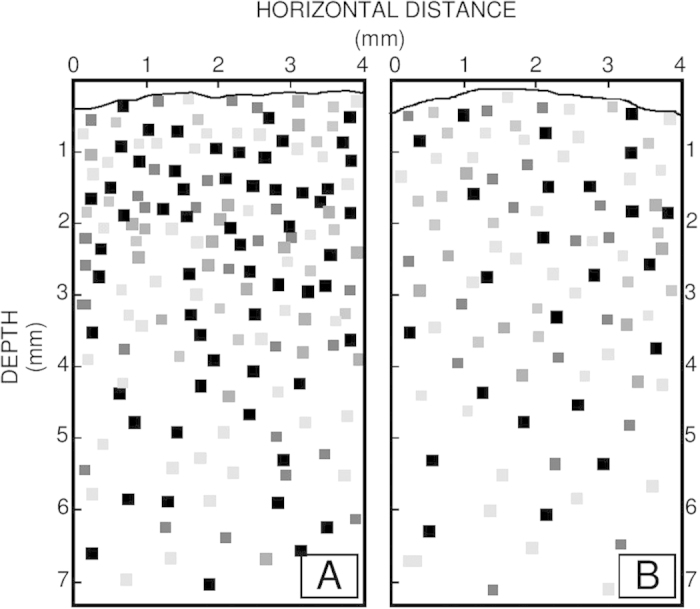
Two-dimensional distribution of sulfate reduction in Shark Bay mats visualized using the silver foil technique. Trace near the top of the panels indicates the surface of the mats. Pixels indicate hotspots of sulfate reduction; darker pixels represent higher rates. (**A**) Smooth mat, showing high rates, especially close to the surface of the mat, coinciding with the zone of supersaturated [O_2_]. (**B**) Pustular mat, displaying a more diffuse pixel pattern, i.e., lower rates and a sulfate reduction distribution pattern that is less concentrated in the oxic zone than was found in the smooth mat.

During the day, below 3 mm depth in smooth mats, sulfide concentrations started to increase with depth, and the concentrations increased at night when sulfide was detected near the surface of the mat. The sulfide concentrations below the oxic zone remained low in the pustular mats and increased slightly during the night (Fig. 3). The difference in sulfide concentration between both mats is again indicative of higher metabolic rates in smooth mats. This was further supported by the ^35^S-silver foil measurements showing high sulfate-reducing activity near the surface, especially in the smooth mats (Fig. 5). The oxic zone was designated as the top 3–3.5 mm in smooth mats and top 5 mm in pustular mats, whereas depths underneath are designated as anoxic zones. As there were increasing levels of sulfide observed at depths 3–8 mm and 6–8 mm, these were designated as H_2_S-rich zones in smooth and pustular mats, respectively.

Overall, the bacterial community in smooth mats was dominated by Proteobacteria (23.5%), Chloroflexi (23.2%), Planctomycetes (12.3%), Cyanobacteria (7.5%), Bacteroidetes (6.6%), Spirochaetes (5.4%), Caldithrix (2.6%), Firmicutes (2.33%), GN04 (1.9%) and OP8 (1.7%). Pustular mats were dominated by Proteobacteria (40.8%), Bacteroidetes (12.8%), Planctomycetes (12.1%), Chloroflexi (11.6%), Cyanobacteria (4.1%), Acidobacteria (3.5%), Spirochaetes (2.5%), GN04 (2%), Verrucomicrobia (2%), Gemmatimonadetes (1.9%) and Actinobacteria (1.5%). Recently published functional metagenomic data supports these groups identified in the present study as being core members of the Shark Bay mat microbiome^23^. Rare, novel candidate bacterial phyla (OP8, GN04, OP3 Caldithrix, TM6, SAR406, OP9, GN02, Hyd24-12, OD1, WS3, BRC1, KSB3) were found in the present investigation, which were not detected in previous clone library studies^8^. These novel bacteria may not have been detected previously due to technological limits, however the present findings suggest that there is a reservoir of potentially undiscovered novel biodiversity in the Shark Bay ecosystem. These novel bacteria comprise a considerable population in the microbial mats (>15% in smooth mats, ~10% in pustular mats) (Supplementary Table 2), representing a range of potentially novel metabolisms in these systems.

Interestingly, smooth mats had detectable levels of sequences affiliated to the lineages OP8, GN01, OP3 and Brocadiae whilst those taxa were absent in pustular mats (Supplementary Fig. S2). In order to test for any statistically significant dissimilarity between the mat types with regard to their community structure, one-way analysis of similarity test (ANOSIM) was carried out. ANOSIM analyses indicated that there were significant differences in microbial community patterns between smooth and pustular mats (Global R = 0.999, *p* = 0.1%). Therefore, this suggests that both mats, although close in physical proximity, have significantly different microbial community structures, which is supported by the differences observed in oxygen and sulfide geochemistry.

### Bacterial community distribution

The photic-oxic zone of smooth mats was highly enriched by cyanobacteria, which occupies 38% of the sequences but diminish drastically below 2 mm (Fig. 2a). One predominant phylotype detected was *Microcoleus*, which represented the cyanobacterial phylum throughout the depths of smooth mats. Other detected cyanobacterial lineages were *Halomicronema* and *Leptolyngbya*, though only in low abundance (Supplementary Fig. S2). In addition to Cyanobacteria, bacterial phyla Proteobacteria and Bacteroidetes not only showed high abundance at the surface, but also displayed uniform distribution with depth. As described in detail later, some members of these groups have known anaerobic lifestyles. Underneath the photic zone, Chloroflexi dominated the smooth mats, which was mainly comprised of the class Anaerolineae (Supplementary Fig. S2). Firmicutes remained a minority except with a notable peak at the bottom layer (18–20 mm). Along with Planctomycetes, these groups were restricted to the anoxic portion of the mats. Alphaproteobacteria was represented in particular by *Dichotomicrobium thermohalophilum* (Supplementary Fig. S2a), an aerobic, chemoorganotrophic bacterium that can thrive in hypersaline environments^26^.

Phototrophic members such as Rhodobacterales (Alphaproteobacteria) were also abundant at the surface. Most of the Deltaproteobacteria were associated with the anaerobic Desulfobacterales and Desulfovibrionales. Surprisingly, the anaerobic sulfate-reducing bacteria comprise 10% of the surface population. Gammaproteobacteria peaked at the surface layer, with most sequences affiliated with Chromatiales (phototrophic purple sulfur bacteria, Supplementary Fig. S2). Brocadiae, a Planctomycetes subphylum lineage that is able to carry out anaerobic ammonia oxidation, was found only in smooth mats. A number of OTUs (up to 5%) clustering with the phylum Caldithrix were only found in the anoxic zone of smooth mats, while this group was virtually absent in pustular mats (<0.1%). Candidate phyla OP8, GN01 and OP3 were found exclusively in smooth mats and restricted to the anoxic zone, suggesting a preference to an anaerobic lifestyle.

In pustular mats, similar bacterial distribution patterns to smooth mats were observed (Supplementary Fig. S3). Alphaproteobacteria, Cyanobacteria and Bacteroidetes were the most abundant lineages at the surface layer. Cyanobacteria were represented predominantly by *Halomicronema* in pustular mats compared to *Microcoleus* in smooth mats. However, unlike smooth mats, Alphaproteobacteria continued to dominate the lower depths instead of Chloroflexi. Furthermore, pustular mats had a lower population of Deltaproteobacteria, Cyanobacteria, Spirochaetes and Firmicutes compared to smooth mats. This could be linked to carbonate precipitation—notably cyanobacteria and sulfate-reducing bacteria (Deltaproteobacteria) play key roles^11^,^28^ – and may be more prevalent in smooth than in pustular mats^27^. Moreover, pustular mats had a higher abundance of Acidobacteria, Actinobacteria and Gemmatimonadetes, with the affiliated sequences increasing with depth (Supplementary Fig. S3).

Verrucomicrobia were discovered at the surface layer, and *Bdellovibrio*, a parasitic Deltaproteobacteria, was only found in pustular mats. Chromatiales, in contrast to smooth mats, represented only half of the Gammaproteobacteria sequences and were well distributed among the mat depths. The lower population of sulfate-reducing bacteria in pustular mats again points at more rigorous sulfur cycling in smooth mats, and is supported by the sulfide depth profiles that indicate a higher rate of both sulfate reduction and putative phototrophic sulfide consumption at the oxygen-sulfide interface at the bottom of the photic zone.

### Phylogenetic clustering with respect to depth

**Figure 6:**
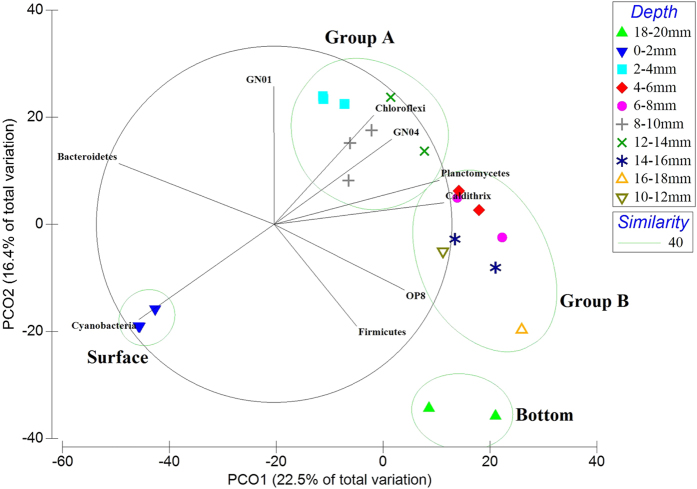
Principal coordinate analysis (PCoA) of Shark Bay smooth mat microbial commumity profiles from different depths. Bray-Curtis similarity matrices of bacterial 16S rRNA gene sequence abundance derived from square-root treatment were used. The green circles represent cluster analysis, which groups layers that share 40% of phylogenetic similarity together at OTU level. PCoA clearly depicts four clustered formed within the smooth mats. The groups were designated surface, group A, group B and bottom. Bacterial phyla with strong correlation (Pearson’s p > 0.7) with respect to depth were overlaid on the PCO plot. Black lines indicate the direction of increased taxon abundance at phylum level, and the length indicates the degree of correlation of the taxa with community data. Cyanobacteria was most abundant at the surface layer, Bacteroidetes is correlated between the surface and group A, Chloroflexi and candidate phylum GN04 are stronlgy correlated to Group A, Planctomycetes showing strong relationship to Group B and Firmicutes linked to the bottom layer.

**Figure 7:**
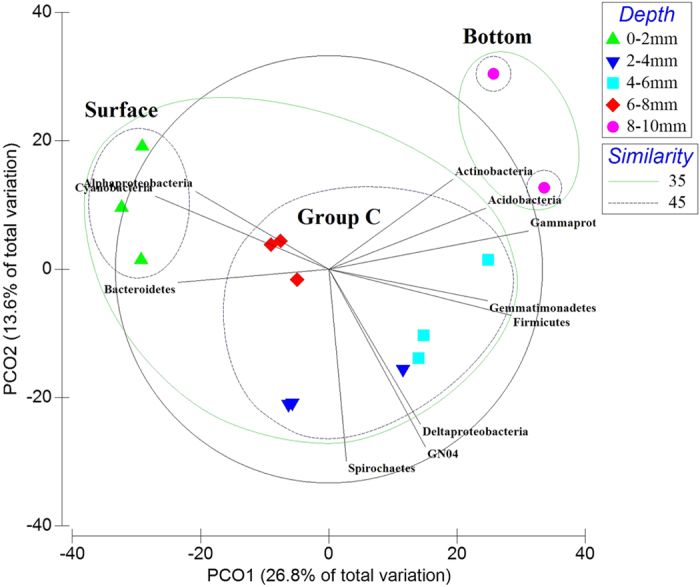
Principal coordinate analysis (PCoA) of Shark Bay pustular mats microbial community profiles from different depths. Results indicated that pustular mat layers were stratified at OTU level into three groups, designated the surface (0–2 mm), Group C (2–8 mm) and the bottom (8–10 mm). Bacterial phyla with strong correlation (Pearson’s p > 0.7) with respect to depth were overlaid on the PCO plot. Black lines indicate direction of increased taxon abundance at phylum level (Class level for Proteobacteria), and the length indicates the degree of correlation of the taxa with community data. Alphaproteobacteria and Cyanobacteria were affiliated with the surface layer, whilst Delta-, Gammaproteobacteria, Spirochaetes and Firmicutes showed very strong correlation to Group C.

Direct comparison of the bacterial mat layer communities by cluster analysis and principle coordinate analysis (PCoA) were undertaken, revealing four clusters for smooth mats and three clusters for pustular mats (Figs 6 and 7), indicating both mats were phylogenetically stratified. Smooth mats were phylogenetically stratified into four distinct clusters (Fig. 6). The groups were designated Group A, Group B, surface, and bottom. The surface layer (0–2 mm) and bottom layer of smooth mats (18–20 mm) formed their own clusters. The layers in between (2–18 mm) were clustered into Group A and Group B. Similarly, pustular mats could be divided into three clusters based on PCO analysis (Fig. 7), namely surface (0–2 mm), Group C as the middle layers (2–8 mm) and bottom (8–10 mm). STAMP analysis revealed that not only qualitatively do Alpha-, Gammaproteobacteria, Cyanobacteria and Bacteroidetes dominate the surface layer of smooth mats, but also statistically these bacterial phyla were over-represented in the surface layer of smooth mats (Supplementary Fig. S4). The middle layers (Group A and B) of smooth mats were characterised by the Chloroflexi class Anaerolineae and phylum Planctomycetes. The bottom layer in smooth mats was significantly enriched with Deltaproteobacteria and OP3. This analysis demonstrated how stratified clusters differ from each other, and which bacteria taxa were over-represented and characterised those clusters.

STAMP analysis of pustular mats revealed that surface layers were statistically characterised by Cyanobacteria and Bacteroidetes, whereas the bottom layers were enriched by various Gammaproteobacteria (Supplementary Fig. S5). Group C of pustular mats did not have any bacterial phyla that were significantly over-represented from the other clusters, suggesting a more even distribution of microorganisms within pustular mats.

### Elemental and network correlation analyses

As chemical elements may link to certain biological activities and specific taxonomic groups^29^, elemental analysis was undertaken on each sample layer using laser ablation with inductively coupled plasma mass spectrometry. The most abundant elements were calcium (>300,000 μg/g), sodium (>10,000 μg/g), magnesium (>4,000 μg/g) and potassium (>2000 μg/g). Elemental analyses did not reveal any distinct patterns based on depth through the mats. Vanadium and magnesium were mainly found in the surface layers, and were more abundant in pustular mats. However, molybdenum was only found in smooth mats, and was either absent in pustular mats or under the detection limits of the LA-ICP-MS (<10 μg/g) employed in the present study.

As ANOSIM has shown that both mats have distinct microbial community structures, it would be informative to determine whether specific elements can be putatively linked to niche formation and differentiation in these ecosystems. To facilitate this characterisation, stepwise distance-based linear model (DistLM) analyses were performed (Table 1). DistLM analysis revealed the four major elements that were significantly (*p* < 0.05) correlated with the variances between microbial communities of smooth and pustular mats, using non-parametric multivariate regression. DistLM indicated that molybdenum appeared to be the most influential element, which alone accounted for 31.66% of the overall variance between the microbial structure of smooth and pustular mats. Molybdenum, iron, manganese and magnesium accounted for nearly half of the overall variance between the microbial community structures of both mats (Table 1). Due to the fact that molybdenum was found exclusively in smooth mats, it is postulated that this particular element may have play a role in the variances of the microbial structures in both mat types.

**Figure 8:**
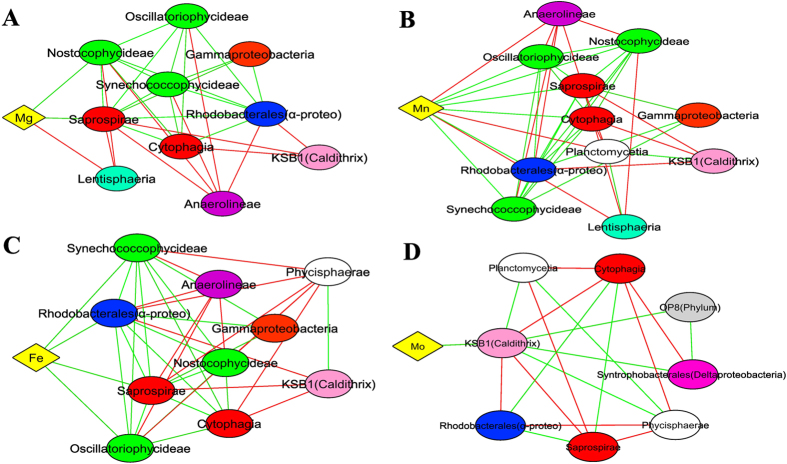
Network correlation analysis between elements (Mg, Mn, Fe and Mo) and bacteria in Shark Bay smooth mats. (**A**) Magnesium. (**B**) Manganese. (**C**) Iron correlation. (**D**) Molybdenum. Connections are given for a strong (Pearson’s p > 0.6) and significant (*p*-value < 0.001) correlations. Each node represents a bacterial class or element. Alpha- and Deltaproteobacteria are presented at the order level. Green lines indicate positive correlations whilst red lines indicate negative correlations. The reoccurring bacterial taxa may indicate overlapped niches. Diamond shape indicates element whilst eclipse shapes represent bacteria. Different colours indicate elements and bacterial phyla. Yellow: element, Green: Cyanobacteria, Blue: Alphaproteobacteria Orange: Gammaproteobacteria, Pink: Deltaproteobacteria, Purple: Chloroflexi, White: Planctomycetes, Light pink: Caldithrix, Light blue: Lentisphaeria, Light grey: OP8.

Network correlation analysis can be used to describe putative direct or indirect interactions between microbial taxa^30^. Such analyses in diverse microbial communities may help to ascertain functional roles or environmental niches occupied by uncultured bacteria^31^,^32^. A highly inter-correlated group was observed in the smooth mats (Supplementary Fig. S6). It consists of a network of Alphaproteobacteria (Order Rhodobacterales), Gammaproteobacteria, Bacteroidetes (Class Cytophagia, Saprosipra), and Cyanobacteria (Class Synechococcophycideae, Oscillatoriophycideae and Nostocophycideae) that are all positively correlated and linked together. As STAMP analysis revealed that all these bacterial classes (with the exception of Nostocophycideae) are over-represented at the surface layer of smooth mats (Supplementary Fig. 4a), it suggests that these clusters may form a consortium at the surface. The cluster was also strongly positively correlated to the elements iron, manganese and magnesium (Fig. 8), elements often found in phototrophic microorganisms^33^. In addition, these bacterial taxa showed negative correlations with anaerobic bacteria such as Anaerolineae and Lentisphaeria. Molybdenum, in contrast, was only positively correlated to Caldithrix. An interconnected cluster of known anaerobic bacteria, including Planctomycetes and Chloroflexi^34^,^35^, were positively correlated with candidate phyla OP3, BRC1 and WS3, suggesting those novel bacterial taxa are likely exhibiting anaerobic metabolism (Supplementary Fig. S6). Furthermore, the distinct clustering of aerobic and anaerobic microorganisms indicated mutual exclusion patterns of each other (Fig. 8a–d).

**Figure 9:**
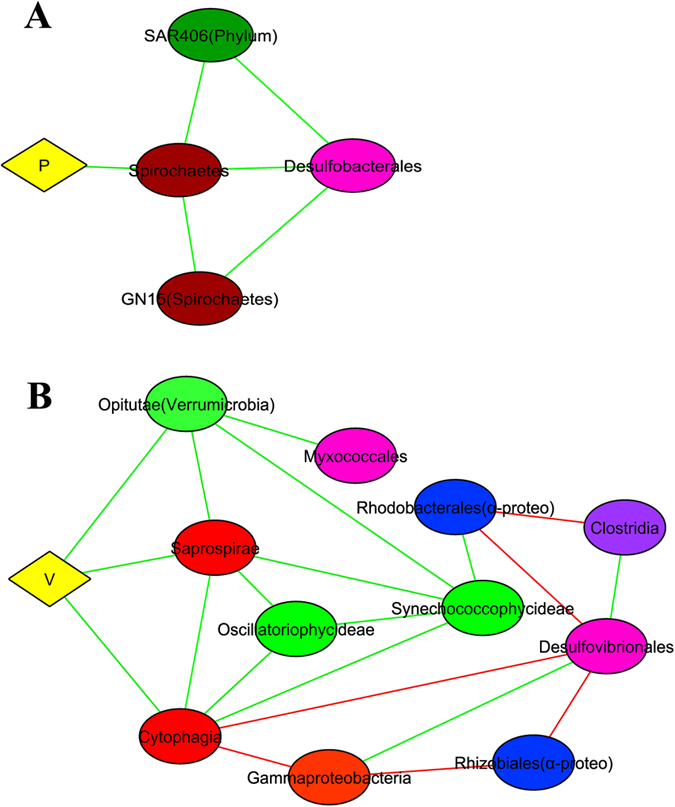
Network correlation analysis between elements (P and V) and bacteria in Shark Bay pustular mats. (**A**) Phosphorus correlation with bacteria, (**B**) Vanadium correlation with bacteria. Connections are given for a strong (Pearson’s p > 0.6) and significant (*p*-value < 0.001) correlations. Each node represents a bacterial class or element. Alpha- and Deltaproteobacteria were presented at the order level. Green lines indicate positive correlations whilst red lines indicate negative correlations. The reoccurring bacterial taxa may indicate overlapped niches. Diamond shape indicates element whilst eclipse shapes represent bacteria. Different colours indicate elements and bacterial phyla. Yellow: element, Green: Cyanobacteria, Red: Bacteroidetes, Blue: Alphaproteobacteria, Orange: Gammaproteobacteria, Pink: Deltaproteobacteria, Brown: Spirochaetes, Light green: Verrucomicrobia, Dark purple: Firmicutes, Dark green: SAR406.

Pustular mats showed similar correlation clusters between Alphaproteobacteria, Bacteroidetes and Cyanobacteria to smooth mats, except that the cluster was negatively correlated to Gammaproteobacteria (Supplementary Fig. S7). An interconnected cluster was found to be associated to anaerobic bacteria (Acidobacteria, Planctomycetes and Gemmatimonadetes), indicating microorganisms were well clustered corresponding to the geochemical gradients of the mats determined through measurements of oxygen and sulfide (Fig. 3). Phosphorus showed positive correlation with Spirochaetes, while Vanadium was positively correlated to Bacteroidetes and Verrucomicrobia (Fig. 9).

## Discussion

This study delineates for the first time the spatial distribution of bacteria with depth in distinct Shark Bay microbial mats, complemented by biogeochemical measurements and elemental analyses. One significant finding of this study was the large proportion of novel bacterial phyla found in these mats. All of these new phyla were not identified in previous studies on these microbial ecosystems except TM6^8^ (Supplementary Table 2). The new sequences substantially increase the representation of these novel candidate bacteria in Shark Bay, and illustrate the power of next generation sequencing to improve our understanding of microbial diversity in these systems. The novel bacteria comprise up to 10–15% of the microbial population, potentially representing (a range of) novel metabolic pathways. Among the novel bacterial phyla, Caldithrix, OP3, OP8 and GN01 were found exclusively in smooth mats. All of these sequences were found in the anoxic zone, demonstrating the potential preference towards a strict anaerobic lifestyle. It has been suggested that OP8 possesses versatile metabolic capabilities^36^,^37^, and some of its members have branched out to form the novel phylum Nitrospirae^17^. Candidate phylum GN01 has also been found in hypersaline mats in Guerrero Negro, Mexico, suggesting a link between microbial mats in disparate geographic locations^22^. It is hypothesized that different biogeochemical environments (i.e., shallow oxygen penetration and higher sulfide concentrations, higher rates of sulfate reduction and O_2_ production and consumption) observed in smooth mats in the present study may contribute to the prevalence of these relatively unique groups. However exactly why these candidate phyla were only found in smooth mats, and their ecological role(s), remains unknown.

Previous studies suggest that metabolite exchange occurs at a micrometer scale in microbial mats, posing a steep biochemical gradient^9^,^38^. Niche differentiation is a concept that describes the tendency for co-existing species to differ in their adaptations to the habitat, and is believed to occur among this gradient to sustain the vast biological diversity of microbial mats^39^. The high microbial diversity observed in the present study is likely to be a result of niche differentiation in the Shark Bay systems. Furthermore, the diel fluctuations of O_2_ and H_2_S, along with the phylogenetically stratified nature of the microbial mats, suggest metabolic cooperation, which leads to niche differentiation.

### Putative phototrophic consortium

The surface layer of smooth mats is dominated by Cyanobacteria, which comprise 40% of sequences. Cyanobacteria are generally considered the main producers of photosynthates, including exopolymeric substances (mucilage) that fuel and protect the mats^9^,^40^, and the high abundance of Cyanobacterial rRNA genes in the surface layer of the mat is consistent with that role. Other known phototrophic or putative phototrophic members of the Alphaproteobacteria (e.g. Rhodobacterales) and Gammaproteobacteria (e.g. Chromatiales) were also localized at the surface, with both representatives containing bacteriochlorophylls and some that are photoheterotrophic, (photolithotrophic or chemolithotrophic) in light^41^. STAMP analysis quantitatively demonstrated that a significantly abundant cluster of Cyanobacteria, Bacteroidetes, Alpha- and Gammaproteobacteria was localized at the surface layer of smooth mats, while pustular mat surfaces were characterised by various Cyanobacteria and Bacteroidetes classes (Supplementary Figs S4a and S5a). Furthermore, network correlation revealed an interconnected cluster consisting of Alphaproteobacteria (Rhodobacterales), Gammaproteobacteria, Cyanobacteria and Bacteroidetes (Cytophagia and Saprospirae) in smooth mats (Supplementary Fig. S6), while similar patterns were observed at the surface of pustular mats, except that Gammaproteobacteria was negatively correlated to the Bacteroidetes-Alphaproteobacteria-Cyanobacteria cluster (Supplementary Fig. S7).

These significant co-occurrences and associations between the bacterial taxa and elements may be used as an indicator of potential niche preferences or synergetic relationships^30^. Empirical evidence from a global ocean metagenomics expedition has shown that Alphaproteobacteria, Gammaproteobacteria and Bacteroidetes are major carriers of proteorhodopsin^42^, a novel photo-enhanced energy-harvesting protein. Proteorhodopsin-based phototrophs represent a potentially unique mode of energy acquisition and carbon assimilation in marine environments^43^,^44^. It is proposed that in this putative phototrophic niche at the mat surface, cyanobacteria and putative proteorhodopsin-carrying Proteobacteria and Bacteroidetes produce organic matter that maintains the heterotroph biomass. Bacteroidetes may also have a putative ecological role of breaking down high molecular weight (MW) macromolecules, including EPS, and cyanobacterial derived metabolites, making organic matter more accessible to the microbial community^23^,^45^. Bacteroidetes are known to have a preference towards metabolising high MW organic matter and complex polysaccharides^46^. It is suggested that Bacteroidetes are associated with Cyanobacteria in close proximity in order to access the high molecular weight organic exudates^47^, as smooth mats may have a surplus of organic carbon on the surface due to the high O_2_ production and consumption rates observed. Therefore the microbial community underneath the surface of mats could gain access to the degraded organic matter, and it is this exploitation of energetic opportunities that likely supports the high microbial diversity in Shark Bay.

### Putative anoxic niche at mat surfaces

As light has limited penetration through microbial mats^9^,^48^, it was not surprising that both mats had enriched phototrophs at the surface, which decreased along with depth (Supplementary Fig. S8). However, there was also an abundance of potential anaerobic fermenters residing at the surface in both mat types. Pustular mats also had a lower abundance of cyanobacteria at the surface compared to smooth mats, and unexpectedly anaerobes were more abundant than phototrophs even at the surface layer in pustular mats. Aerobic heterotrophs and sulfate-reducing bacteria (SRB) were well distributed throughout smooth mats, however the obligately anaerobic SRB were found even at the oxic surface layers (10% of OTUs in smooth mats, 5% of OTUs in pustular mats). The majority of the detected representatives of the Deltaproteobacteria in Shark Bay were *Desulfococcus* and *Desulfovibrio*, suggesting sulfate reduction even at the oxic surface layer. The presence of SRB has been reported for several mats with smooth surfaces and high metabolic activity of the entire community^18^,^19^,^49^. The finding of SRB in the oxic zone of the mat was supported by data in the present study on sulfate-reduction in the mats (Fig. 5), in addition to a recent study whereby sulfate-reducing activity was also observed at the oxic surface layer in smooth mats in Shark Bay^15^. It is speculated that the anaerobic fermenters at the surface may have a role in providing low molecular weight organic carbon and H_2_ to the SRB. Thus it is suggested that an anoxic niche exists in the surface mat matrix to protect SRB from oxidative stress. The distribution of SRB in microbial mats forming marine stromatolites in the Bahamas was mapped using Geographical Information Systems and concluded clusters of SRB were common in lithifying mats^50^. Furthermore, our data indicating a large portion of other anaerobic members at the surface oxygenated layer (e.g. Anaerolineae, Spirochaetes, Planctomycetes, Deltaproteobacteria) provides further support for the existence of an anoxic niche in the photic-oxic zone of Shark Bay microbial mats, or alternatively, some possess oxygen-insensitive metabolisms or survival strategies^51^.

As alluded to earlier, one potential ecological role of the surface anoxic niche might be mat lithification. Sulfate reduction had also been found in the oxygenated zones in a diverse range of microbial mats^19^, such as hypersaline mats in Guerrero Negro, Solar Lake, Kiritimati Atoll, and Bahamas^11^,^18^,^52^,^53^. SRB that are localised in the photic-oxic zone have been linked to carbonate precipitation at the mat surface^24^,^28^,^54^. The coupling of SRB activities and cyanobacteria may be linked to mat lithification (net carbonate precipitation)^3^. The recent identification of specific niches of sulfide production coinciding with cyanobacterial photosynthesis^15^, further supports the proposal here of metabolic cooperation in modern microbialites, especially as it relates to the higher lithification potential proposed for smooth mats than for pustular mats^27^.

### Putative spectral photoheterotrophic niche under photic zones

In hypersaline environments, energy-expensive osmotic regulation is required for microbial survival, thus anaerobic microorganisms that have low energy-yielding metabolisms should theoretically be eliminated due to bioenergetics constraints^55^. Although some studies have demonstrated that hypersalinity can limit microbial diversity^56^,^57^, our study indicates a highly diverse microbiota. Given both the high diversity and abundance of putative anaerobic heterotrophs in both mat types (Supplementary Fig. S8), it suggests an ecological model in Shark Bay where niche differentiation may facilitate tightly coupled microbial interactions.

Interestingly, as vibrations of water molecules and the surface coarse sediment can scatter light, a series of distinct spectral niches can be generated along the light spectrum even under photic zones^58^,^59^. As both mats examined in the present study are situated in the intertidal zone in Shark Bay, daily disturbance of tidal waves may potentially contribute to creating distinct spectral niches, thus facilitating a significantly different microbial structure in smooth and pustular mats. Indeed, conceptually it seems unlikely for phototrophs to occur deep in the mat, however an unexpected trend of increasing cyanobacteria (e.g. *Microcoleus* sp.) was observed below the oxic zone at 5–20 mm depth in smooth mats in the present study, suggesting the potential for a distinct spectral niche deep in the mat. There has been the report of a cyanobacterium isolated from Shark Bay stromatolites containing a novel chlorophyll *f*, which can absorb light at the infrared part of the spectrum^60^. Given the fact that infrared light can penetrate deeper into microbial mats^48^, it is possible that cyanobacteria deep in the smooth mats potentially possess these (or other) novel chlorophylls, where the red-shifted pigment could extend the phototrophic light spectrum. *Microcoleus* has also been shown to switch from an oxygenic to anoxygenic phototrophic lifestyle under anoxia with high levels of H_2_S^61^,^62^. However it should be noted that *Microcoleus* in particular has been shown to migrate vertically in a diel cycle^63^,^64^,^65^, and also demonstrates alternate metabolisms such as fermentation of endogenous carbohydrates under dark, anoxic conditions^66^,^67^.

A recent study has found phototrophic Gemmatimonadetes in geographically dispersed environments containing carotenoids as the phototrophic pigment^68^, suggesting this poorly described phylum as photoheterotrophic. Thus Gemmatimonadetes found in pustular mats may potentially be putative phototrophic and form a distinct spectral phototrophic niche in the anaerobic zone. The potential phototrophic niche below the photic zone may have an ecological role for “filling the niches”, increasing the efficiency of energy production and nutrient cycling, allowing microbial co-existence and diversity through niche creation^59^,^69^.

### *A H_2_S-molybdenum paradox?*

Interestingly, using the methods employed in the current study, molybdenum (Mo) was only detected in smooth mats. The predominant form of Mo in typical marine habitats is the molybdate salt^70^. Studies have demonstrated that the presence of molybdate inhibits sulfate respiration, hence restricting H_2_S concentrations in microbial mats^19^,^71^,^72^,^73^. However, *in situ* microelectrode measurements undertaken in the present investigation indicated that smooth mats have more than twice the concentration of sulfide than pustular mats over depths of 4–8 mm (Fig. 3), and higher sulfate reduction rates (Fig. 5). Thus the co-existence of a H_2_S-rich zone and Mo leads to a potential sulfide-Mo paradox in smooth mats. It is possible that Mo may not manifest itself in the form of molybdate in the Shark Bay mats, or there is a specific niche protecting SRB from molybdate inhibition. Furthermore, Mo detected in smooth mats might be in the form of molybdoenzymes (e.g. nitrogenase, sulfite oxidase)^74^,^75^, and certain species of SRB can produce MoSO_2_^76^ which potentially contributes to the higher detectable levels of both Mo and sulfide observed in smooth mats. However, further work is needed to clarify which Mo species is present and whether Mo has any defining roles in the Shark Bay microbial mat systems.

### Putative nitrogen fixation niches in smooth mats

Although cyanobacteria are thought to be the primary nitrogen fixers in microbial mats, research has shown direct coupling of nitrogen fixation with sulfate reduction in microbial mats and marine sediments^77^,^78^,^79^. In our study, STAMP analyses revealed that bottom mat layers are characterised by Deltaproteobacteria, and studies in deep-sea systems have shown that nitrogen-fixing Deltaproteobacteria are often coupled with methane-oxidising archaea in a syntrophic relationship^80^. Furthermore, horizontal gene transfer of the nitrogenase gene has been observed globally, whereby nitrogenase gene sequences were found in *Clostridium* Firmicutes, Delta- and Gammaproteobacteria^81^. The sharp peak of Firmicutes at the bottom layer in smooth mats (Supplementary Fig. S2g), along with the increasing trend of *Microcoleus* and the abundance of Deltaproteobacteria, suggests a potential nitrogen-fixing niche in the lower depths of smooth mats. Consortial N_2_ fixation is the suggested symbiotic strategy in smooth mats where microorganisms cooperate to enhance nitrogen cycling^82^,^83^. Mo is a co-factor of nitrogenase, which may potentially facilitate the formation of a nitrogen fixation niche in smooth mats^74^.

Of further relevance to nitrogen cycling, network correlation analyses indicated that Mo was only correlated to the phylum Caldithrix (Fig. 8d), and this bacterial phylum was only found in smooth mats. This novel phylum is known to be involved in the nitrogen cycle by performing nitrate reduction^84^, and may play such a role in the smooth mats. Interestingly, typical nitrifying bacteria (i.e. *Nitrosomonas, Nitrobacter*) were not detected in both mats in the present study. This is consistent with a recent metagenomic study, where it was suggested an alternative pathway of nitrification may be present in these systems^23^, potentially filled by ammonia-oxidising archaea. Further work on archaeal diversity and distribution in the Shark Bay mats is required to ascertain the exact role(s) of this domain in nitrogen cycling.

### Putative nitrogen fixation and methanogenic/methanotrophic niches in pustular mats

Network correlation and elemental analyses in pustular mats demonstrated that vanadium is positively correlated to surface Bacteroidetes and Verrumicrobia (Fig. 9b). In addition, elemental analyses revealed that pustular mats have a higher concentration of vanadium and the levels peak at the surface. Although a recent study demonstrated that some Bacteroidetes are able to carry out nitrogen fixation^85^, it is not known if this is the case in Shark Bay.

However, as Mo was not detected in pustular mats, nitrogen fixation in this system could potentially be performed in part by Mo-independent nitrogenase, such as iron or vanadium nitrogenase^86^,^87^. Taken together, this suggests a putative nitrogen fixation niche at the surface in pustular mats, although there is the potential for vanadium to be associated with other metabolisms. Interestingly, it has been shown that Mo-independent nitrogen fixation is coupled with a significant amount of H_2_ as a by product^88^. The large quantity of H_2_ produced is suggested to maintain a population of methanogenic archaea in pustular mats^89^. Although SRB also reside at the mat surface and it has been shown that SRB thermodynamically outcompete methanogens^90^,^91^, the use of non-competitive substrates^21^ or spatial compartmentalisation may allow the coexistence of SRB and methanogens. Furthermore, the excess amount of photosynthetically originated organic carbon derived from the steep O_2_ gradients observed in the present study may support methanogenesis at the surface^21^. Verrucomicrobia are more abundant in pustular mat surfaces and known to exhibit aerobic methanotrophy^92^,^93^, which could potentially metabolise methane produced by the putative archaeal community in pustular mats. Therefore it is suggested that there is a putative nitrogen fixation niche at the pustular mat surface, coupled with a putative methanogenic and methanotrophic niche.

### Potential evolutionary significance of smooth mats

The presence of Mo in smooth mats may have potential evolutionary implications. Marine Mo concentrations were less than one-tenth of modern values in the Precambrian, where Mo-nitrogen co-limitation may have influenced the early evolution of cyanobacteria^94^. Thus, there was likely strong selection for bacteria with strategies for Mo acquisition and retention during the early development of microbial mats. Furthermore, enrichment of Mo is also observed in Guerrero Negro microbial mats^95^, and it is proposed that elevated levels of Mo can be used as a geochemical tool to indicate conditions associated with the presence of microbial mats in ancient hypersaline environments^73^,^95^.

### Potential ecological significance of niche differentiation in Shark Bay mats

The dominance of the Chloroflexi class Anaerolineae in smooth mats but not in pustular mats, along with the novel candidate bacterial phyla found exclusively in smooth mats may be a result of niche differentiation. Metabolic specialisation of a range of microorganisms in different putative niches might have contributed to the dominance of Anaerolineae in smooth mats, and shaped the assembly of distinct microbial communities^96^. Furthermore, pustular mats are located closer to the shore along a tidal transect compared to smooth mats^27^, where potentially higher desiccation, temperature, and salinity stress could occur. Thus as pustular mats are exposed to these extremes for longer periods, this may have contributed to the different geochemical gradients and hence microbial differentiation observed in the present study. In addition to some expected overlap, metabolic versatility may also allow microbes to be present in different mat layers, contributing to the heterogeneous nature of the mats. In contrast, metabolic specialisation may allow switching metabolic pathways in specific depths. For example, *Microcoleus* observed in the present study may switch from oxygenic phototrophy to anoxygenic phototrophy in deeper anoxic layers. Metabolic speciation and synergies may also be potentially moderated by quorum sensing in Shark Bay mats, as suggested in Bahamian microbialites^97^. Shark Bay mats may represent a unique ecological model containing a wide spectrum of autotroph-heterotroph interactions ranging from mutualistic nitrogen fixation niches and phototrophic niches, to opportunistic niches that exploit every energetic opportunity to maintain the microbial diversity observed.

## Conclusions

This is the first study to characterise bacterial distribution at a millimeter scale in Shark Bay microbial mats, employing high throughput sequencing platforms. Elemental analyses have provided insights into potential links with the prevailing biota, although further work is needed to definitively conclude whether a given element is indeed being sequestered by a particular biological process, or whether geochemical processes are influencing their enrichment. It is postulated that niche differentiation may occur and can help our understanding of how microbial communities can survive and adapt in environments of high salinity, UV radiation, desiccation and diel fluctuation of biogeochemical gradients. Although this study has provided key insights into the spatial distribution of bacteria with potential niches postulated, as alluded earlier delineating the depth distribution of archaea in particular will be important to fully characterise these communities. Seasonal analyses may also facilitate building potential models of microbial succession in these systems. Another caveat is that 16S rDNA data can be limited in facilitating exact links with proposed functions, and thus recent metagenomic studies^23^, as well as future work examining defined gene expression in specific organismal groups in Shark Bay, will allow for robust conclusions to be drawn on the complex interactions proposed here. Nonetheless this study has enabled a holistic view of these ecosystems, and facilitated a greater understanding of the complex network of putative niches and microbial interactions in modern microbial mats.

## Materials and Methods

### Sample collection and *in situ* microelectrode measurements

Smooth and pustular mats were sampled at 1500 h on 16^th^ June 2013 from Nilemah, Hamelin Pool, Shark Bay (26°27′336′′S, 114°05.762′′E), using methods as previously described^5^. At the time of collection, the water temperature was 24.8 °C, salinity 68 PSU, and pH 8.13. Mat samples were placed in sterile containers and immediately preserved in RNALater (Ambion, Life Technologies) by completely immersing the samples. In addition to sample collection, metabolic profiles (oxygen and sulfide) were measured for the smooth and pustular mats over a 24-h cycle. These *in situ* measurements were taken using microelectrodes as described previously^11^,^15^,^98^, where needles probes were used to record diel fluctuations of oxygen and sulfide concentrations with depth. Immediately following sampling of mats, the rates of oxygen production and consumption were measured using the light/dark shift approach^11^,^38^ *ex situ*. Vertical mat sections were cut and incubated on ^35^SO_4_^2−^-labeled silver foil to determine sulfate reduction rates in the upper ~5 mm of the mats^28^. The light intensity was determined using a LiCor LI 250 meter equipped with a SA190A quantum sensor. Salinity and temperature measurements were obtained with an Accumet AP75 temperature/conductivity meter and pH of the overlying water and the sediment at 0–10 mm depth were determined with a Hannah handheld pH-meter.

### Sample description and sectioning

Smooth mats (Fig. 1A) were characterised by flat, smooth surfaces with a beige colour and a green layer at the surface. Peloids and ooids were found in the samples, with a few white bivalve shells trapped between the sediment grains. Small carbonate grains, indicative of *in situ* precipitation were sometimes present in a semi-continuous layer at the interface of the caramel/green and black layers. Pustular mats (Fig. 1B) were characterised by black surfaces of gelatinous pustules composed of mucilage, with successive layers underneath. Generally, these mats contain trapped and bound grains, and rarely show pockets of carbonate mineral precipitation. In order to determine the microbial composition in successive layers of the microbial mat, the samples were dissected into 2 mm intervals using sterilised blades. Smooth mat samples were measured to be 20 mm from top to bottom, whereas pustular mat samples had a 10 mm thickness from top to bottom (Fig. 1). Each microbial mat layer was separated into two aliquots: one for DNA extraction/sequencing, and the other for elemental analysis.

### Elemental analysis

To semi-quantitatively determine elemental concentration in the mats, laser ablation with inductively coupled plasma mass spectrometer (LA-ICP-MS) was employed. Sectioned mat samples (0.5 g for each layer) were analysed in duplicate at the Mark Wainwright Analytical Centre, UNSW. The surface of the samples was removed by laser ablation to minimise surface contamination. The entire ablation process was carried out in an inert gas chamber with helium and argon acting as the carrier gas^99^. The ablated samples were then sent through ICP-MS for elemental analysis.

### Nucleic acid extraction

Total environmental DNA was extracted in duplicate from each microbial mat layer (10 layers from smooth mat, 5 from pustular mat) employing the MoBio PowerBiofilm DNA Isolation Kit (MO BIO Laboratories, Carlsbad, USA) according to the manufacturer’s instructions. The concentrations and purity of extracted DNA were determined spectrometrically, and the quality checked by PCR amplification of bacterial 16S rRNA genes^5^.

### 16S rDNA tag sequencing and analysis

Paired-End sequencing of bacterial 16S rRNA genes was performed on an Illumina MiSeq desktop sequencer as described^100^. Amplicon production was performed using barcoded 27F/519R primers that targeted the V1 to V3 region of the bacterial 16S rRNA gene. Amplicon primers were 27F (5′TATGGCGAGTGA AGAGTTTGATCMTGGCTCAG-3′) and 519R (5′-CAAGCAGAAGACGGCATACGAGATXXXXXXXXXXXXAGTCAGTCAGGG GWATTACCGCGGCKGCTG-3′). Sequence reads longer than 500 bp, containing ambiguous nucleotides and having homopolymers longer than 8 bp, were removed using the Mothur software package version 1.33.0. The sequences were then aligned (Needleman-Wunsch pairwise alignment) with the Silva Bacteria Database^101^ version 115, and filtered to remove non-informative columns. Chimeric sequences were removed using UCHIME included in MOTHUR^102^ in *de novo* mode. After chimeras were removed, sequences were classified using the Wang approach as the default settings in Mothur against the Greengenes database (August 2013 version)^103^. Subsequently, rRNA gene sequences from each group were randomly subsampled to 50,000 sequences (lowest number among the dataset). Sequences were then clustered *de novo* into operational taxonomic units (OTU) at a genetic divergence level of 3%, which represents species-level^104^. The OTUs were assigned with taxonomy using the classified sequences generated above. Taxonomic richness and diversity estimators were calculated using MOTHUR^105^. Sequences have been deposited in MG-RAST under accession numbers 4604139.3 to 4604190.3.

### Statistical analyses

One-way Analysis of similarity test (ANOSIM) program was employed to examine whether the microbial communities between both mat types were significantly different. Cluster analysis of communities was also performed based on the resemblance matrix constructed. Principal coordinate analysis (PCoA) undertaken was plotted and clustered groups were overlaid on PCO graphs. PCoA was performed based on matrices derived from the phylum level, and variables with strong correlation (Pearson’s *p* > 0.6) were overlaid on PCO plots to indicate how different bacterial phyla putatively correlate to different depths in the mats. To further explore microbial stratification, statistical analysis of metagenomic profiles (STAMP) was unertaken^106^. Stratified groups were compared to all others to identify which bacterial taxa in each group were best discriminated significantly between other designated groups. Welch’s t-test was employed to reduce type I errors^107^, and Benjamini-Hochberg false discovery rate correction was applied as a multiple-hypothesis test correction. All quoted *P*-values represent corrected values (equating to *q*), with values <0.05 considered significant^108^. A filter was applied to remove taxa with a *q* value > 0.05.

The statistical program R v 2.14.0 with the *Hmisc* and *Igraph* library package was used for generating correlation networks between microbial taxa and element concentrations^109^. In order to show only strong correlations, bacterial phyla and elements having weak correlations (Pearson’s p < 0.6, *p* > 0.001) were excluded from the analysis. A correlation network was built where nodes represent either a bacterial taxon or element. Correlation network graphs were exported to Gephi version 0.8.2, then visualised using Cytoscape version 3.1.1^110^. Element abundance was also incorporated into PCO plots to indicate putative correlations of elements with microbial groups. Only elements that were above the LA-ICP-MS detection limit (>10 μg/g) were selected. Stepwise distance-based linear model analysis (DistLM) was used to model the relationship between element concentrations and its contribution to the variation in microbial community structure^108^. ANOSIM, PCoA and DistLM were all undertaken in the PRIMER 6 plus Permanova software package. Bray-Curtis similarity measures were used to produce the resemblance matrices.

## Additional Information

**How to cite this article**: Lun Wong, H. *et al.* Niche differentiation of bacterial communities at a millimetre scale in Shark Bay microbial mats. *Sci. Rep.* **5**, 15607; doi: 10.1038/srep15607 (2015).

## Supplementary Material

Supplementary Information

## Figures and Tables

**Table 1:** **Distanced based linear model (DISTLM) on elemental analysis.**

Variable^a^	*F*^b^	*P*^c^	Cumul.^d^
Mo	15.29	0.001	31.66%
Fe	4.7935	0.001	40.57%
Mn	3.0769	0.001	45.93%
Mg	2.2503	0.001	49.71%

Molybdenum, iron, manganese and magnesium accounted for 50% of the overal variances in the microbial structure between smooth and pustular mats.

^a^Four major elements which were significantly (*p* < 0.05) correlated with the variances between microbial communities of smooth and pustular mats, using non-parametric multivariate regression

^b^Pseudo *F-*values (*F*).

^c^*P*-values (*P*).

^d^Cumulative percentage of variance explained
