# Supplementary material for: Niche differentiation of bacterial communities at a millimeter scale in Shark Bay microbial mats
Source: Sci Rep. 2015 Oct 26;5:15607. doi: 10.1038/srep15607 (PMC4620479; doi:10.1038/srep15607)
Supplement: Supplementary Information [file srep15607-s1.pdf]

Niche differentiation of bacterial communities at a millimeter scale in Shark Bay microbial mats

Hon Lun Wong [1,3], Daniela-Lee Smith[1,3], Pieter T. Visscher[2,3], Brendan P. Burns[1,3]

[1]School of Biotechnology and Biomolecular Sciences, The University of New South Wales, Sydney, Australia

[2]Department of Marine Sciences, University of Connecticut, USA

[3]Australian Centre for Astrobiology, University of New South Wales Sydney, Australia

*Corresponding author:

Dr Brendan P. Burns

School of Biotechnology and Biomolecular Sciences, University of New South Wales, Sydney, 2052, Australia.

Phone: 612 93853659.

Fax: 612 93851591.

Email: brendan.burns@unsw.edu.au

Subject category: Microbial ecology and functional diversity of natural habitats

Supplementary Figure S1. Rarefaction curves of smooth and pustular mats layers. The bottom layer of smooth mats (SD10, 19 – 20 mm) has the highest number of different OTUs, followed by the 6th layer of smooth mats (SD6, 11 – 12 mm) and the bottom layer of pustular mats (PD5, 9 – 10 mm). P1 to P5 refer to pustular mat layers 1 - 5, while S1 – S10 refer to smooth mat layers 1 – 10.

Supplementary Figure S2. Abundance of some prominent phyla and taxonomic subgroups by depth in smooth mats. **A**. Proteobacteria, **B**. Cyanobacteria, **C**. Chloroflexi, **D**. Bacteroidetes, **E**. Planctomycetes, **F**. Spirochaetes, **G**. Firmicutes, **H**. Other low abundance phyla

Supplementary Figure S3. Abundance of some prominent phlya and taxonomic subgroups by depth in pustular mats. **A**. Proteobacteria, **B**. Cyanobacteria, **C.** Chloroflexi, **D.** Bacteroidetes, **E**. Planctomycetes, **F**. Spirochaetes and Firmicutes, **G**. Other low abundance phyla

Supplementary Figure S4. STAMP analysis of taxonomy enriched or depleted between the designated groups and all other groups in smooth mats. **A.** Surface group compared to the other three groups, showing the surface of smooth mats is characterised by Cyanobacteria, Bacteroidetes, Alpha- and Gammaproteobacteria (Green dots). **B.** Group A compared to all other groups, showing Group A is represented by class Anaerolineae and classes of Spirochaetes (Blue dots) **C.** Group B compared to all other three groups, showing Group B is characterised mainly by Planctomycetes (Red dots). **D.** Bottom layer compared to all other groups, showing the bottom layer is represented by mainly Deltaproteobacteria and candidate phylum OP3 (Brown dots). All tests were performed using Welch's t-tests and subjected to Benjamini-Hochberg FDR correction.

Supplementary Figure S5. STAMP analysis of taxonomy enriched or depleted between the designated groups and all other groups in pustular mats. **A.** Surface group compared to the other two groups, showing the surface of smooth mats is characterised by Cyanobacteria and Bacteroidetes (Green dots). **B.** Bottom layer of pustular mats compared to all other groups, showing the bottom layer is represented Gammaproteobacteria (Red dots) All tests were performed using Welch's t-tests and subjected to Benjamini-Hochberg FDR correction. Group C cluster in pustular mats showed no bacteria taxa that were significantly enriched or depleted.

Supplementary Figure S6. Network correlation analysis at the bacterial class level of smooth mats. Connections are given for a strong (Pearson's p > 0.6) and significant (*p*-value < 0.001) correlations. Diamond shaped nodes represent elements whilst eclipse shaped nodes represent bacterial classes. Alpha- and Deltaproteobacteria were presented at the order level. Green lines indicate positive correlations whilst red lines indicate negative correlations. There are 48 nodes and 141 edges in the network. Different colours indicate elements and bacterial

phyla. Element nodes in yellow represents correlation with bacteria whilst element nodes in white indicates no correlation with bacteria. Green: Cyanobacteria, Red: Bacteroidetes, Blue: Alphaproteobacteria, Orange: Gammaproteobacteria, Pink: Deltaproteobacteria, Purple: Chloroflexi, White: Planctomycetes, Light pink: Caldithrix, Light blue: Lentisphaeria, Light grey: OP8, Brown: Spirochaetes, Dark purple: Firmicutes, Dark green: Others (Bacterial phyla OP3, GN04, TM6, WS3, BRC1, Fibrobacteres and SAR406).

Supplementary Figure S7. Network correlation analysis at the bacterial class level of pustular mats. Connections are given for a strong (Pearson's $p > 0.6$) and significant (*p*-value < 0.001) correlations. Diamond shaped nodes represent elements whilst eclipse shaped nodes represent bacterial classes. Alpha- and Deltaproteobacteria were presented at the order level. Green lines indicate positive correlations whilst red lines indicate negative correlations. There are 42 nodes and 139 edges in the network. Different colours indicate elements and bacterial phyla. Element nodes in yellow represents correlation with bacteria whilst element nodes in white indicates no correlation with bacteria. Green: Cyanobacteria, Red: Bacteroidetes, Blue: Alphaproteobacteria, Orange: Gammaproteobacteria, Pink: Deltaproteobacteria, Purple: Chloroflexi, White: Planctomycetes, Brown: Spirochaetes, Light green: Verrucomicrobia, Dark purple: Firmicutes, Dark red: Gemmatimonadetes, Dark green: Others (Bacterial phyla Actinobacteria, Acidobacteria, Fibrobacteres and SAR406).

Supplementary Figure S8. Spatial distribution and relative abundance of putative bacterial functional groups in Shark Bay microbial mats. A. Smooth mats **B.** Pustular mats. Functional groups were only assigned to those bacterial taxa for which conserved functions are well described.

Additional Information regarding to Supplementary Figure S6 & S7

Supplementary Figure S6:

48 nodes

141 edges

Rhodobacterales (Alphaproteobacteria) have the most edges (13)

Elements having the most edges with bacteria: Manganese (9), Iron (4), Magnesium (3), Molybdenum (1).

Supplementary Figure S7:

42 nodes

139 edges

Rhizobiales (Alphaproteobacteria) have the most edges (6)

Elements having the most edges with bacteria: Vanadium (3), Phosphorus (1)

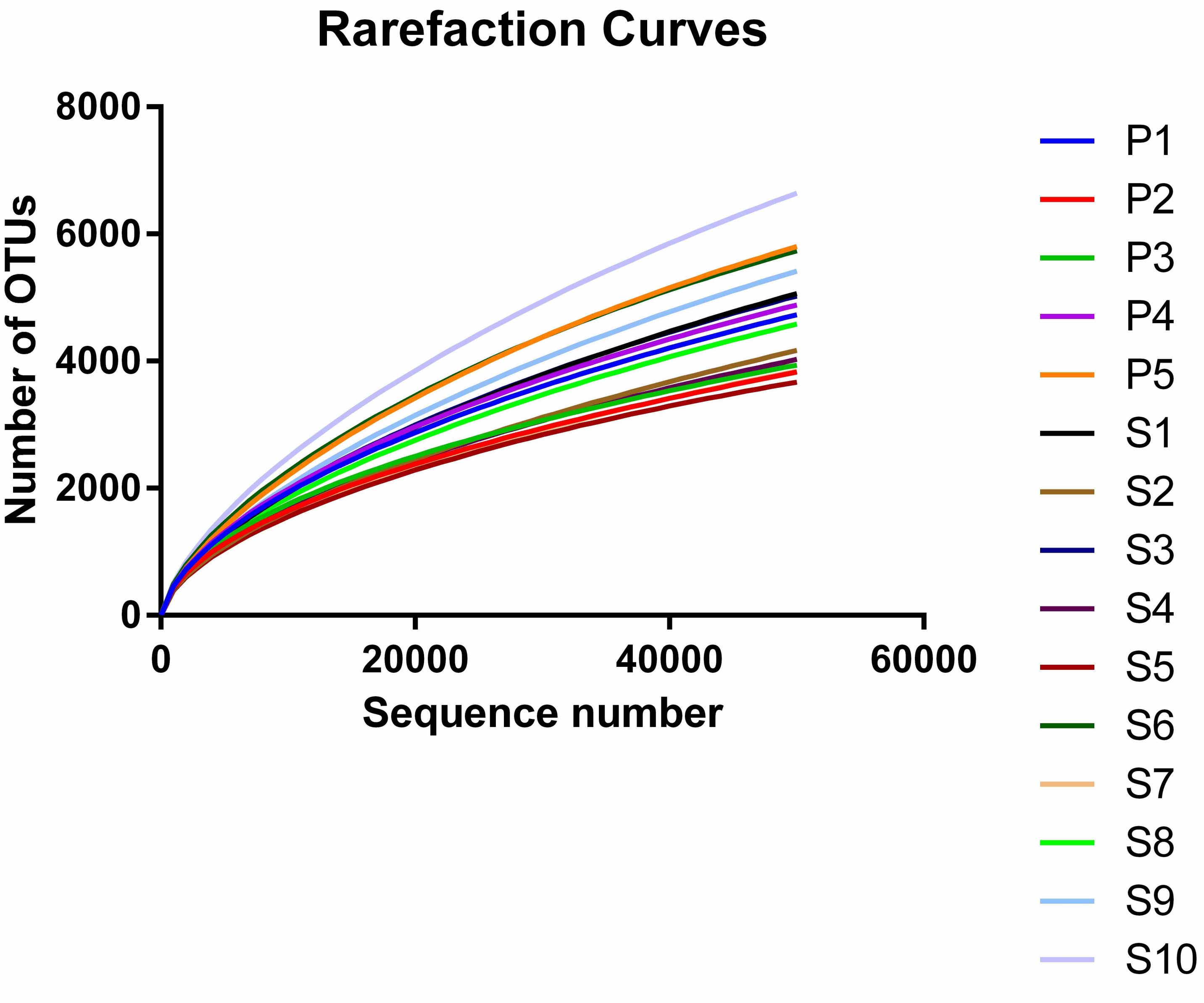

Rarefaction Curves
Number of OTUs
Sequence number
0
2000
4000
6000
8000
0
20000
40000
60000
P1
P2
P3
P4
P5
S1
S2
S3
S4
S5
S6
S7
S8
S9
S10

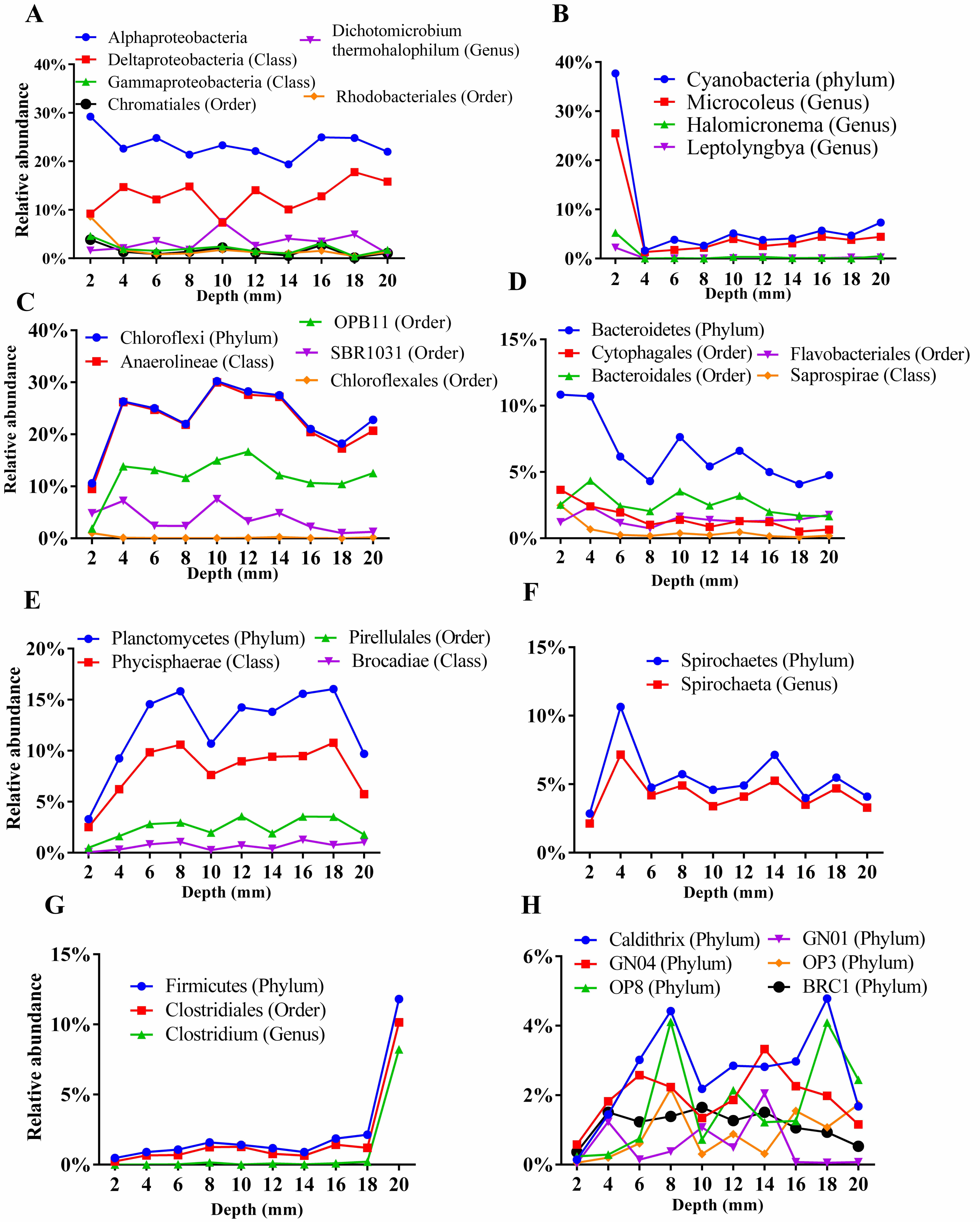

A
Alphaproteobacteria
Deltaproteobacteria (Class)
Gammaproteobacteria (Class)
Chromatiales (Order)
Dichotomicrobium thermohalophilum (Genus)
Rhodobacteriales (Order)
Relative abundance
Depth (mm)
B
Cyanobacteria (phylum)
Microcoleus (Genus)
Halomicronema (Genus)
Leptolyngbya (Genus)
Depth (mm)
C
Chloroflexi (Phylum)
Anaerolineae (Class)
OPB11 (Order)
SBR1031 (Order)
Chloroflexales (Order)
Relative abundance
Depth (mm)
D
Bacteroidetes (Phylum)
Cytophagales (Order)
Bacteroidales (Order)
Flavobacteriales (Order)
Saprospirae (Class)
Depth (mm)
E
Planctomycetes (Phylum)
Phycisphaerae (Class)
Pirellulales (Order)
Brocadiae (Class)
Relative abundance
Depth (mm)
F
Spirochaetes (Phylum)
Spirochaeta (Genus)
Depth (mm)
G
Firmicutes (Phylum)
Clostridiales (Order)
Clostridium (Genus)
Relative abundance
Depth (mm)
H
Caldithrix (Phylum)
GN04 (Phylum)
OP8 (Phylum)
GN01 (Phylum)
OP3 (Phylum)
BRC1 (Phylum)
Depth (mm)

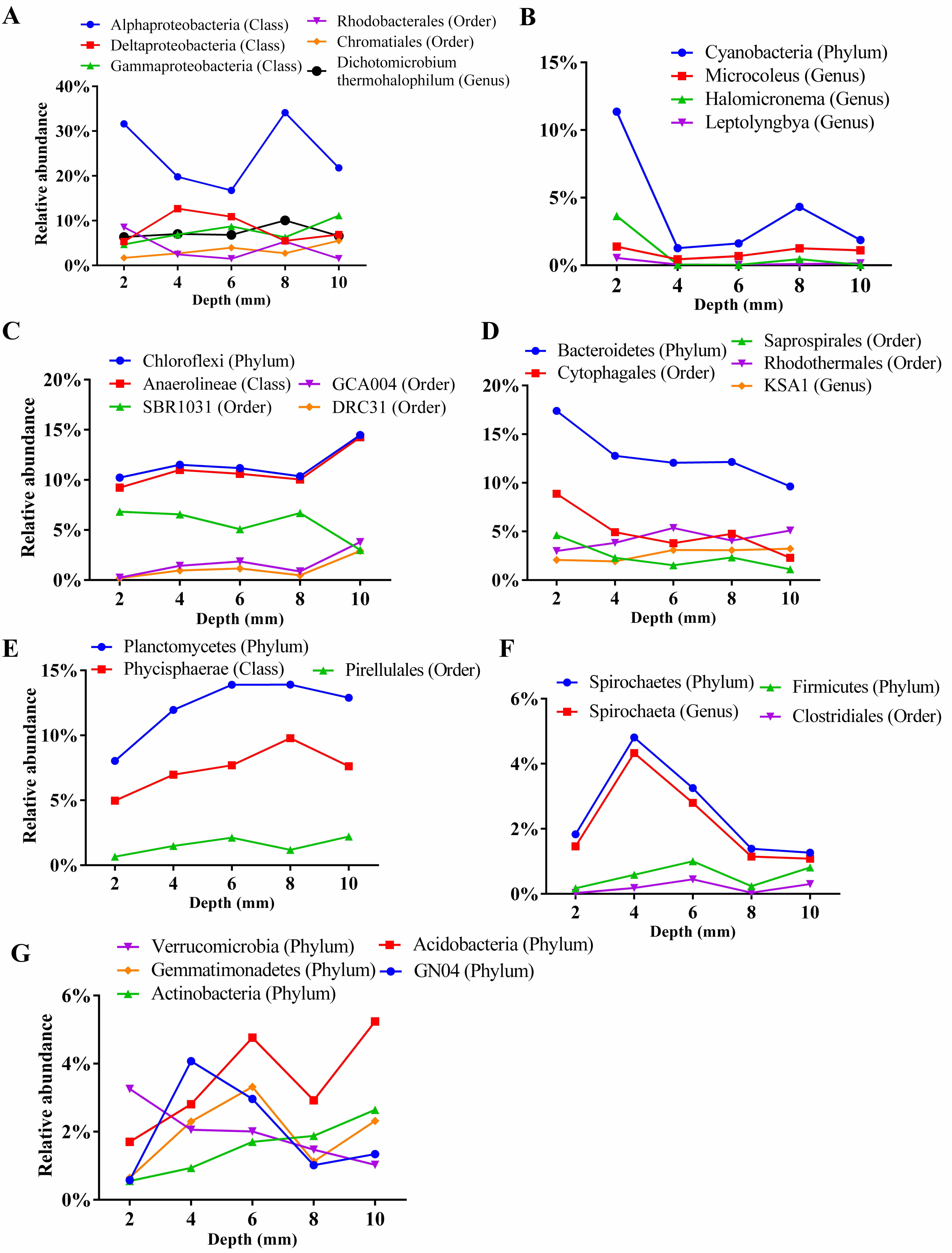

A
Alphaproteobacteria (Class)
Deltaproteobacteria (Class)
Gammaproteobacteria (Class)
Rhodobacterales (Order)
Chromatiales (Order)
Dichotomicrobium thermohalophilum (Genus)
Relative abundance
Depth (mm)
B
Cyanobacteria (Phylum)
Microcoleus (Genus)
Halomicronema (Genus)
Leptolyngbya (Genus)
Depth (mm)
C
Chloroflexi (Phylum)
Anaerolineae (Class)
SBR1031 (Order)
GCA004 (Order)
DRC31 (Order)
Relative abundance
Depth (mm)
D
Bacteroidetes (Phylum)
Cytophagales (Order)
Saprospirales (Order)
Rhodothermales (Order)
KSA1 (Genus)
Depth (mm)
E
Planctomycetes (Phylum)
Phycisphaerae (Class)
Pirellulales (Order)
Relative abundance
Depth (mm)
F
Spirochaetes (Phylum)
Spirochaeta (Genus)
Firmicutes (Phylum)
Clostridiales (Order)
Depth (mm)
G
Verrucomicrobia (Phylum)
Gemmatimonadetes (Phylum)
Actinobacteria (Phylum)
Acidobacteria (Phylum)
GN04 (Phylum)
Relative abundance
Depth (mm)

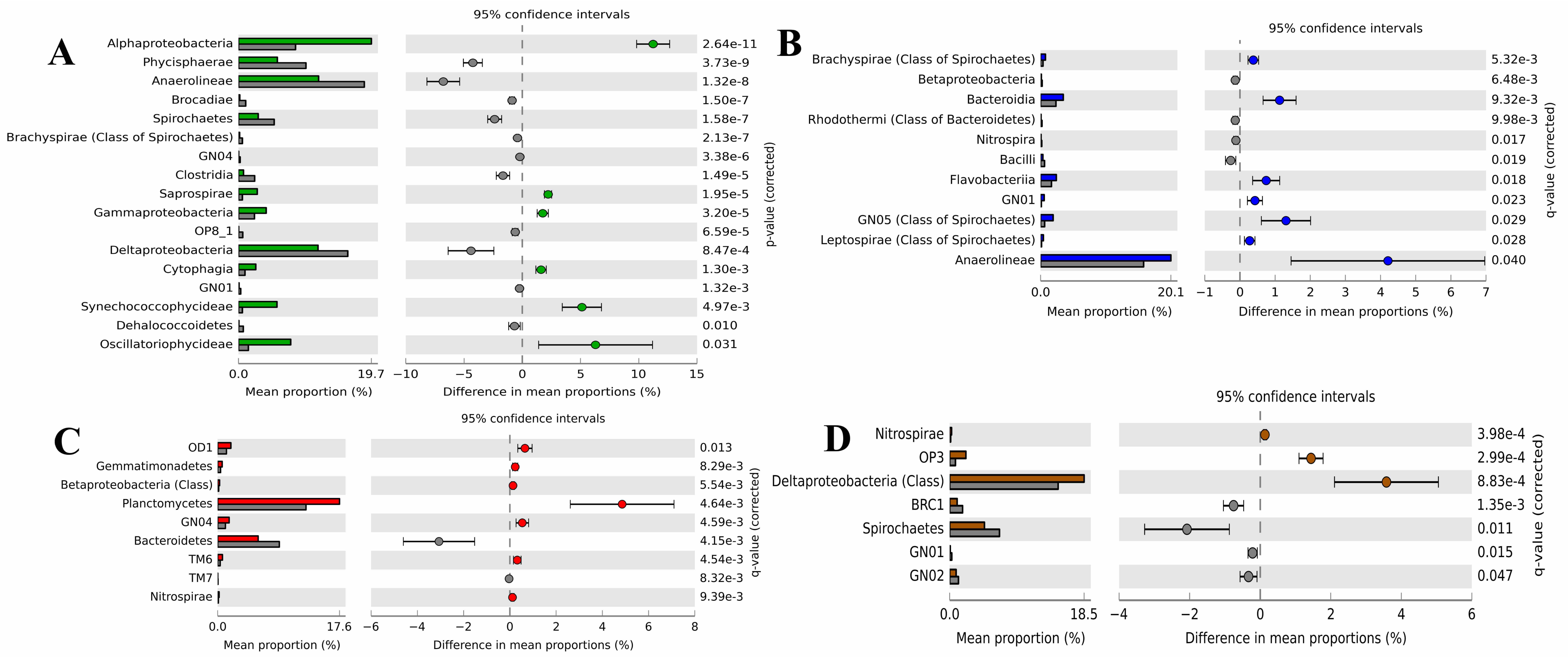
A
95% confidence intervals
Alphaproteobacteria 2.64e-11
Phycisphaerae 3.73e-9
Anaerolineae 1.32e-8
Brocadiae 1.50e-7
Spirochaetes 1.58e-7
Brachyspirae (Class of Spirochaetes) 2.13e-7
GN04 3.38e-6
Clostridia 1.49e-5
Saprospirae 1.95e-5
Gammaproteobacteria 3.20e-5
OP8_1 6.59e-5
Deltaproteobacteria 8.47e-4
Cytophagia 1.30e-3
GN01 1.32e-3
Synechococcophycideae 4.97e-3
Dehalococcoidetes 0.010
Oscillatoriophycideae 0.031
Mean proportion (%)
Difference in mean proportions (%)
p-value (corrected)
B
95% confidence intervals
Brachyspirae (Class of Spirochaetes) 5.32e-3
Betaproteobacteria 6.48e-3
Bacteroidia 9.32e-3
Rhodothermi (Class of Bacteroidetes) 9.98e-3
Nitrospira 0.017
Bacilli 0.019
Flavobacteriia 0.018
GN01 0.023
GN05 (Class of Spirochaetes) 0.029
Leptospirae (Class of Spirochaetes) 0.028
Anaerolineae 0.040
Mean proportion (%)
Difference in mean proportions (%)
q-value (corrected)
C
95% confidence intervals
OD1 0.013
Gemmatimonadetes 8.29e-3
Betaproteobacteria (Class) 5.54e-3
Planctomycetes 4.64e-3
GN04 4.59e-3
Bacteroidetes 4.15e-3
TM6 4.54e-3
TM7 8.32e-3
Nitrospirae 9.39e-3
Mean proportion (%)
Difference in mean proportions (%)
q-value (corrected)
D
95% confidence intervals
Nitrospirae 3.98e-4
OP3 2.99e-4
Deltaproteobacteria (Class) 8.83e-4
BRC1 1.35e-3
Spirochaetes 0.011
GN01 0.015
GN02 0.047
Mean proportion (%)
Difference in mean proportions (%)
q-value (corrected)

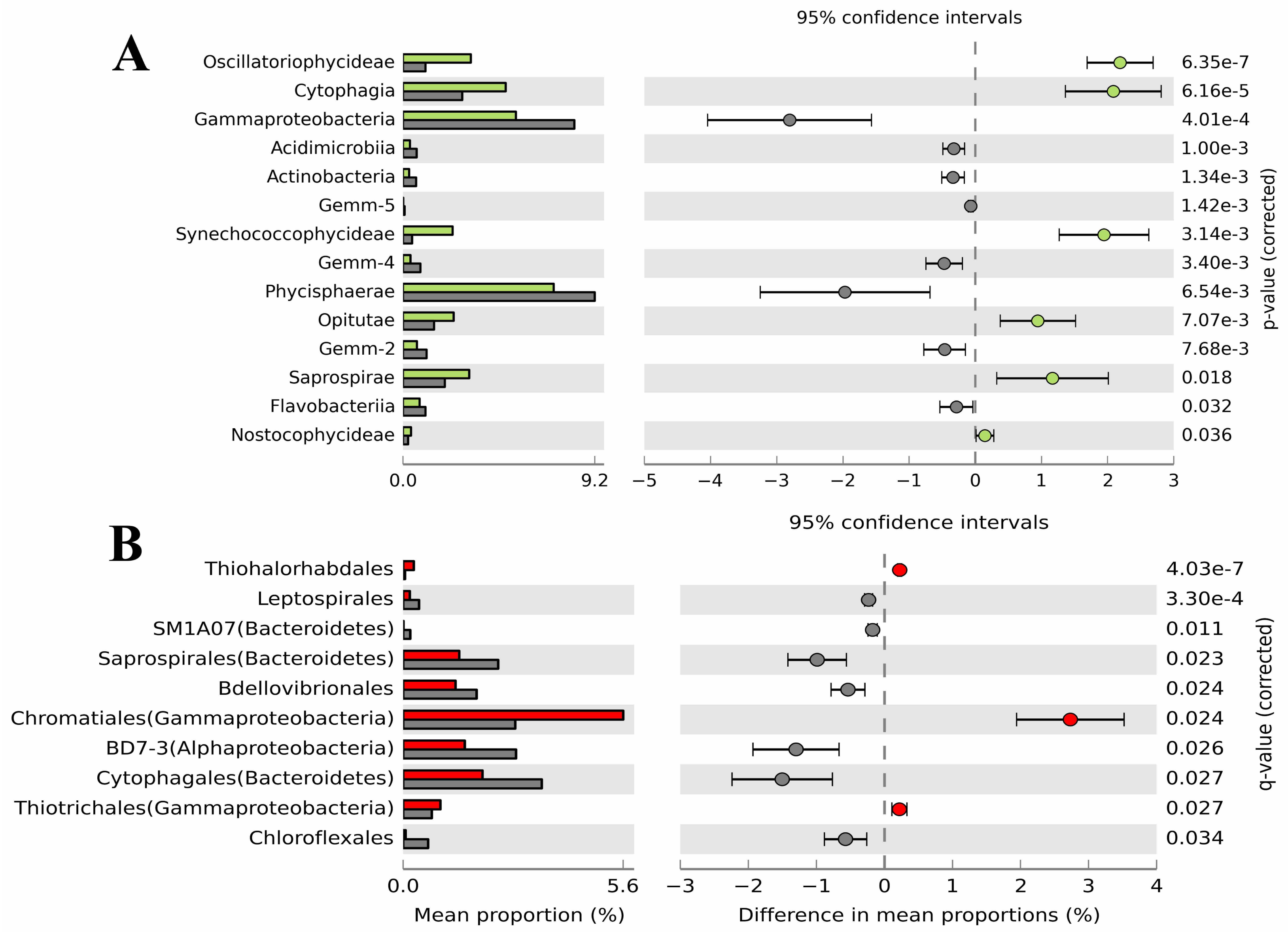
A
95% confidence intervals
Oscillatoriophycideae
Cytophagia
Gammaproteobacteria
Acidimicrobiia
Actinobacteria
Gemm-5
Synechococcophycideae
Gemm-4
Phycisphaerae
Opitutae
Gemm-2
Saprospirae
Flavobacteriia
Nostocophycideae
6.35e-7
6.16e-5
4.01e-4
1.00e-3
1.34e-3
1.42e-3
3.14e-3
3.40e-3
6.54e-3
7.07e-3
7.68e-3
0.018
0.032
0.036
p-value (corrected)
0.0
9.2
−5
−4
−3
−2
−1
0
1
2
3
B
95% confidence intervals
Thiohalorhabdales
Leptospirales
SM1A07(Bacteroidetes)
Saprospirales(Bacteroidetes)
Bdellovibrionales
Chromatiales(Gammaproteobacteria)
BD7-3(Alphaproteobacteria)
Cytophagales(Bacteroidetes)
Thiotrichales(Gammaproteobacteria)
Chloroflexales
4.03e-7
3.30e-4
0.011
0.023
0.024
0.024
0.026
0.027
0.027
0.034
q-value (corrected)
0.0
5.6
−3
−2
−1
0
1
2
3
4
Mean proportion (%)
Difference in mean proportions (%)

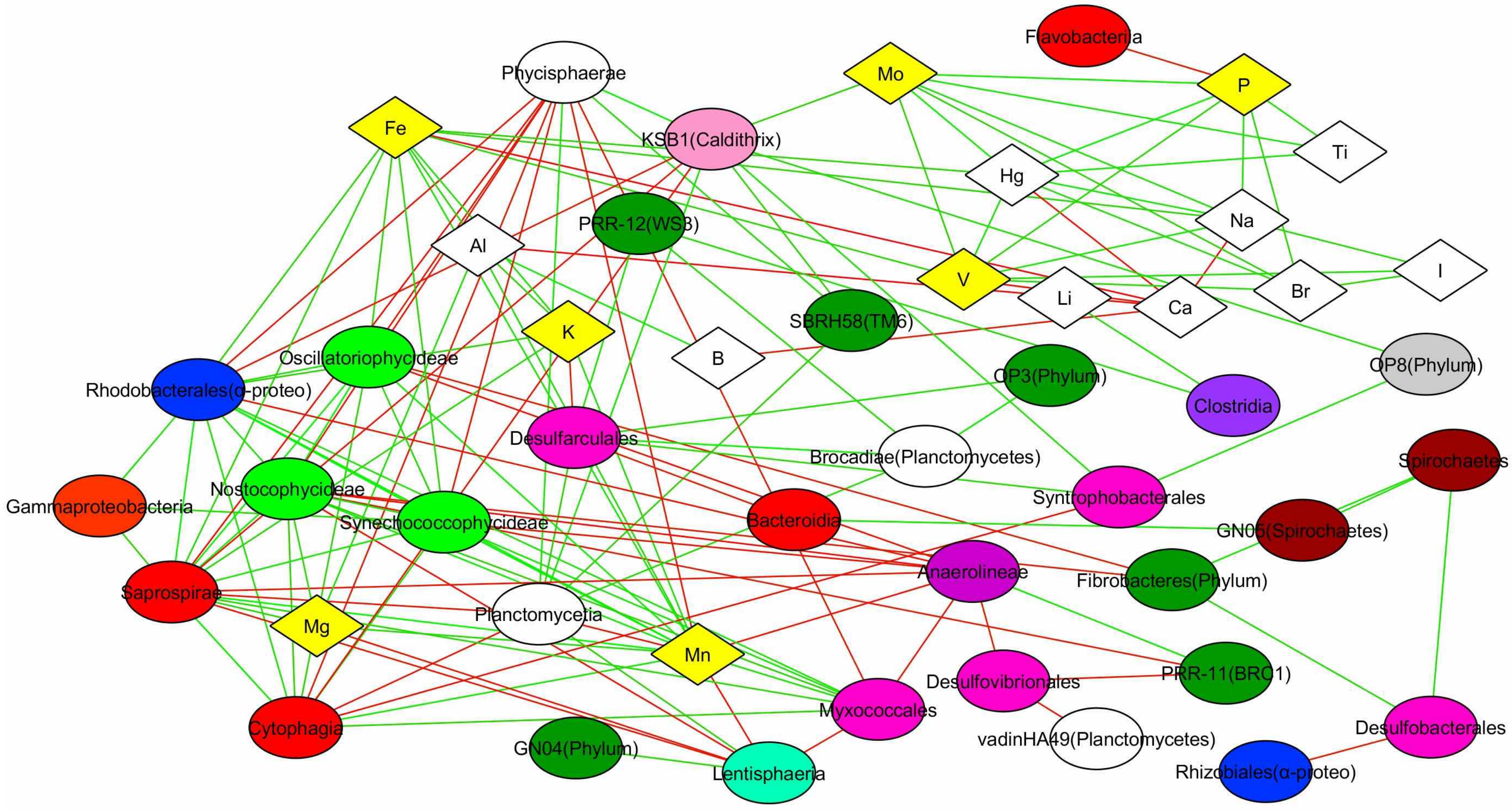
Flavobacteria
Mo
P
Phycisphaerae
Fe
KSB1(Caldithrix)
Ti
Hg
PRR-12(WS3)
Na
Al
V
I
Li
Ca
Br
SBRH58(TM6)
K
B
OP3(Phylum)
Clostridia
OP8(Phylum)
Oscillatoriophycideae
Rhodobacterales(α-proteo)
Desulfarculales
Brocadiae(Planctomycetes)
Spirochaetes
Nostocophycideae
Syntrophobacterales
Gammaproteobacteria
Synechococcophycideae
Bacteroidia
GN05(Spirochaetes)
Anaerolineae
Saprospirae
Fibrobacteres(Phylum)
Planctomycetia
Mg
Mn
Desulfovibrionales
PRR-11(BRC1)
Myxococcales
Cytophagia
vadinHA49(Planctomycetes)
Desulfobacterales
GN04(Phylum)
Lentisphaeria
Rhizobiales(α-proteo)

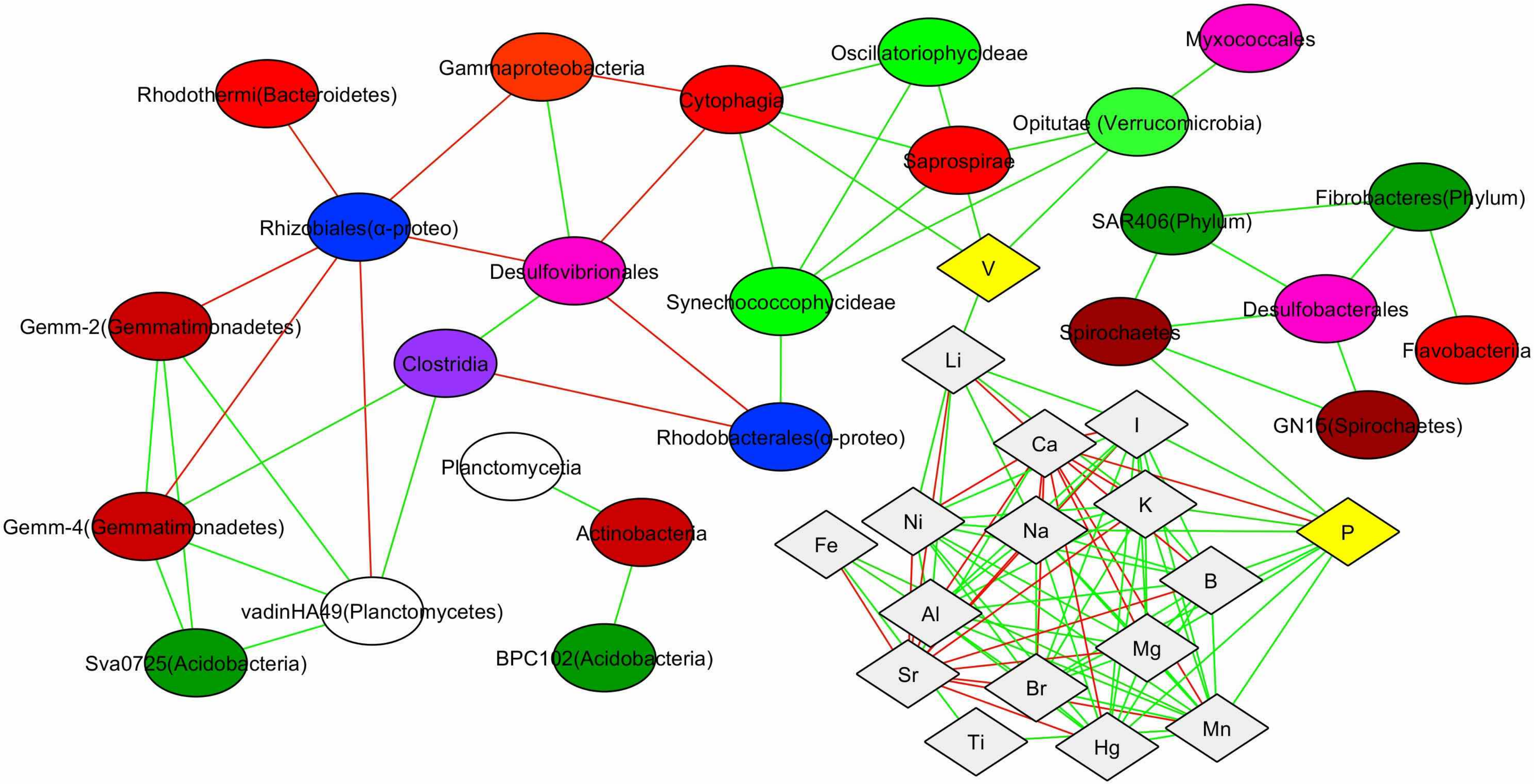

Rhodothermi(Bacteroidetes)
Gammaproteobacteria
Cytophagia
Oscillatoriophycideae
Myxococcales
Opitutae (Verrucomicrobia)
Saprospirae
Rhizobiales(α-proteo)
Desulfovibrionales
Synechococcophycideae
V
SAR406(Phylum)
Fibrobacteres(Phylum)
Desulfobacterales
Spirochaetes
Flavobacteria
Gemm-2(Gemmatimonadetes)
Clostridia
Li
Rhodobacterales(α-proteo)
GN15(Spirochaetes)
Planctomycetia
Ca
I
Ni
Na
K
Fe
P
Gemm-4(Gemmatimonadetes)
Actinobacteria
B
Al
vadinHA49(Planctomycetes)
Mg
Sva0725(Acidobacteria)
BPC102(Acidobacteria)
Sr
Br
Ti
Hg
Mn

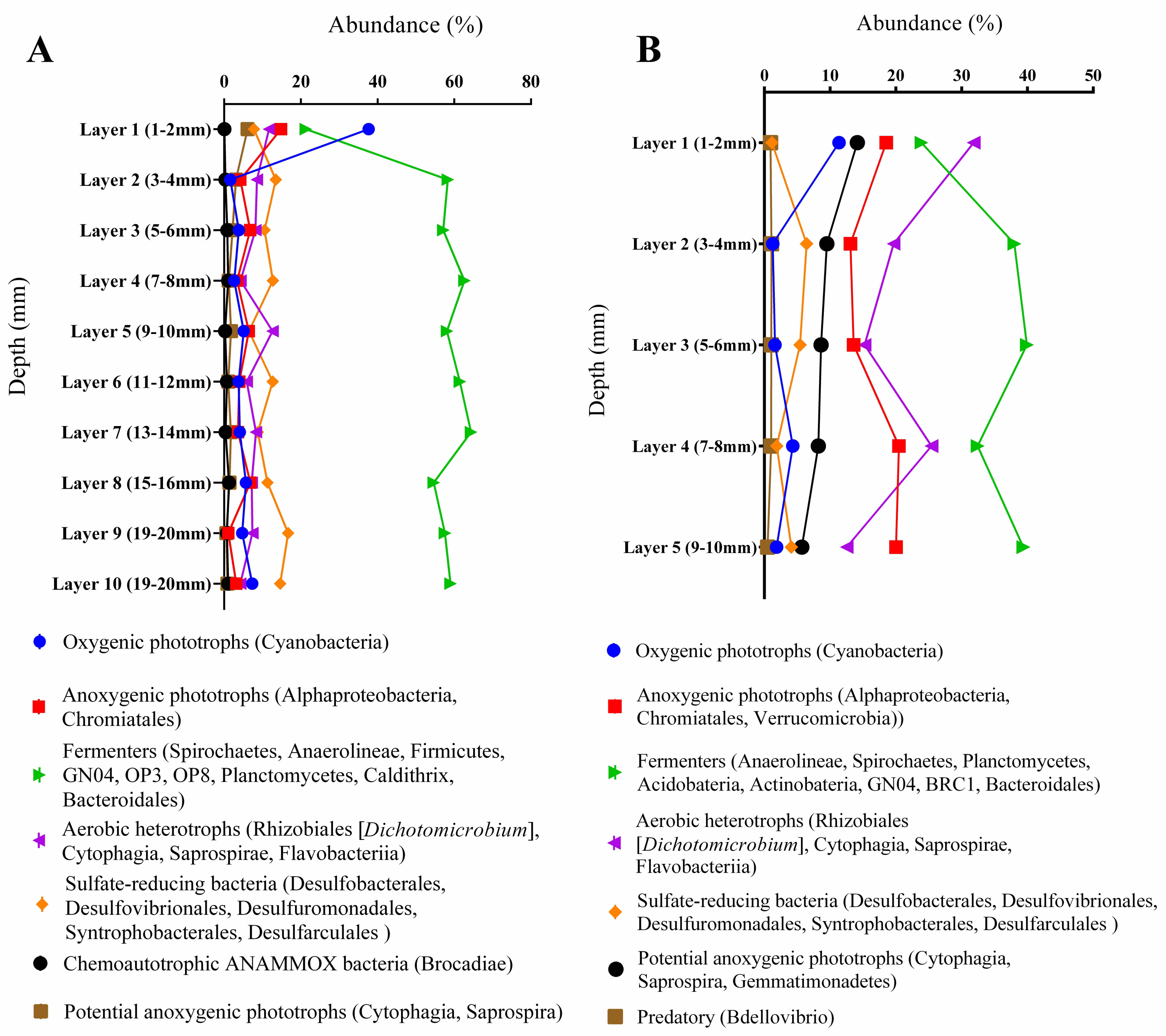
A
Abundance (%)
0
20
40
60
80
Depth (mm)
Layer 1 (1-2mm)
Layer 2 (3-4mm)
Layer 3 (5-6mm)
Layer 4 (7-8mm)
Layer 5 (9-10mm)
Layer 6 (11-12mm)
Layer 7 (13-14mm)
Layer 8 (15-16mm)
Layer 9 (19-20mm)
Layer 10 (19-20mm)
Oxygenic phototrophs (Cyanobacteria)
Anoxygenic phototrophs (Alphaproteobacteria, Chromiatales)
Fermenters (Spirochaetes, Anaerolineae, Firmicutes, GN04, OP3, OP8, Planctomycetes, Caldithrix, Bacteroidales)
Aerobic heterotrophs (Rhizobiales [Dichotomicrobium], Cytophagia, Saprospirae, Flavobacteriia)
Sulfate-reducing bacteria (Desulfobacterales, Desulfovibrionales, Desulfuromonadales, Syntrophobacterales, Desulfarculales)
Chemoautotrophic ANAMMOX bacteria (Brocadiae)
Potential anoxygenic phototrophs (Cytophagia, Saprospira)
B
Abundance (%)
0
10
20
30
40
50
Depth (mm)
Layer 1 (1-2mm)
Layer 2 (3-4mm)
Layer 3 (5-6mm)
Layer 4 (7-8mm)
Layer 5 (9-10mm)
Oxygenic phototrophs (Cyanobacteria)
Anoxygenic phototrophs (Alphaproteobacteria, Chromiatales, Verrucomicrobia))
Fermenters (Anaerolineae, Spirochaetes, Planctomycetes, Acidobateria, Actinobateria, GN04, BRC1, Bacteroidales)
Aerobic heterotrophs (Rhizobiales [Dichotomicrobium], Cytophagia, Saprospirae, Flavobacteriia)
Sulfate-reducing bacteria (Desulfobacterales, Desulfovibrionales, Desulfuromonadales, Syntrophobacterales, Desulfarculales)
Potential anoxygenic phototrophs (Cytophagia, Saprospira, Gemmatimonadetes)
Predatory (Bdellovibrio)

Supplementary Table 1. Bacterial 16S rRNA gene diversity analyses* of smooth and pustular mats

Mat layers	Depth	Good's Coverage[a]	No. OTUs	Chao1[b]	Shannon[c]
Pustular layer 1	1 - 2 mm	0.951	4731	8641	6.361
Pustular layer 2	3 - 4 mm	0.961	3827	7434	6.130
Pustular layer 3	5 - 6 mm	0.962	3939	7333	6.271
Pustular layer 4	7 - 8 mm	0.950	4884	8574	6.335
Pustular layer 5	9 - 10 mm	0.941	5802	9063	6.259
Smooth layer 1	1 - 2 mm	0.944	5063	9479	5.602
Smooth layer 2	3 - 4 mm	0.953	4168	8328	6.105
Smooth layer 3	5 - 6 mm	0.947	5027	8518	6.383
Smooth layer 4	7 - 8 mm	0.958	4028	7340	6.130
Smooth layer 5	9 - 10 mm	0.965	3671	5781	5.905
Smooth layer 6	11 - 12 mm	0.944	5738	8705	6.556
Smooth layer 7	13 - 14 mm	0.960	3846	6894	6.005
Smooth layer 8	15 -16 mm	0.952	4583	8198	6.336
Smooth layer 9	17 - 18 mm	0.940	5418	9727	6.227
Smooth layer 10	19 - 20 mm	0.926	6646	12171	6.581

*Calculated at 3% genetic divergence
[a]Good's Coverage measures how well the sample represents the larger environment
[b]Chao1 index measures the species richness (absolute number of species)
[c]Shannon index measures the species diversity (relative species abundance and species eveness)

Supplementary Table 2. Novel candidate bacteria phyla and bacterial phyla that are poorly described, found in Hamelin Pool ecosystems.

Novel bacterial phyla	Pustular	Smooth
Caldithrix	0.01%	2.95%
GN04	2%	1.91%
OP8	N/A	1.73%
GN01	N/A	0.56%
OP3	N/A	0.89%
Fibrobacteres	0.83%	1.05%
TM6	0.34%	0.72%
SAR406	0.42%	0.27%
BRC1	0.66%	1.14%
Gemmatimonadetes	1.94%	0.45%
WS3	0.21%	0.70%
GN02	0.29%	1.22%
OD1	0.34%	1.25%
Verrumicrobia	1.97%	0.54%
Lentisphaerae	0.49%	0.51%
NKB19	0.42%	N/A
KSB3	N/A	0.48%
Hyd24-12	N/A	0.11%
WS1	N/A	0.11%
OP9	N/A	0.10%
Proportion of population	9.92%	16.69%
